# MicroRNA Related Polymorphisms and Breast Cancer Risk

**DOI:** 10.1371/journal.pone.0109973

**Published:** 2014-11-12

**Authors:** Sofia Khan, Dario Greco, Kyriaki Michailidou, Roger L. Milne, Taru A. Muranen, Tuomas Heikkinen, Kirsimari Aaltonen, Joe Dennis, Manjeet K. Bolla, Jianjun Liu, Per Hall, Astrid Irwanto, Keith Humphreys, Jingmei Li, Kamila Czene, Jenny Chang-Claude, Rebecca Hein, Anja Rudolph, Petra Seibold, Dieter Flesch-Janys, Olivia Fletcher, Julian Peto, Isabel dos Santos Silva, Nichola Johnson, Lorna Gibson, Zoe Aitken, John L. Hopper, Helen Tsimiklis, Minh Bui, Enes Makalic, Daniel F. Schmidt, Melissa C. Southey, Carmel Apicella, Jennifer Stone, Quinten Waisfisz, Hanne Meijers-Heijboer, Muriel A. Adank, Rob B. van der Luijt, Alfons Meindl, Rita K. Schmutzler, Bertram Müller-Myhsok, Peter Lichtner, Clare Turnbull, Nazneen Rahman, Stephen J. Chanock, David J. Hunter, Angela Cox, Simon S. Cross, Malcolm W. R. Reed, Marjanka K. Schmidt, Annegien Broeks, Laura J. V. a. n't. Veer, Frans B. Hogervorst, Peter A. Fasching, Michael G. Schrauder, Arif B. Ekici, Matthias W. Beckmann, Stig E. Bojesen, Børge G. Nordestgaard, Sune F. Nielsen, Henrik Flyger, Javier Benitez, Pilar M. Zamora, Jose I. A. Perez, Christopher A. Haiman, Brian E. Henderson, Fredrick Schumacher, Loic Le Marchand, Paul D. P. Pharoah, Alison M. Dunning, Mitul Shah, Robert Luben, Judith Brown, Fergus J. Couch, Xianshu Wang, Celine Vachon, Janet E. Olson, Diether Lambrechts, Matthieu Moisse, Robert Paridaens, Marie-Rose Christiaens, Pascal Guénel, Thérèse Truong, Pierre Laurent-Puig, Claire Mulot, Frederick Marme, Barbara Burwinkel, Andreas Schneeweiss, Christof Sohn, Elinor J. Sawyer, Ian Tomlinson, Michael J. Kerin, Nicola Miller, Irene L. Andrulis, Julia A. Knight, Sandrine Tchatchou, Anna Marie Mulligan, Thilo Dörk, Natalia V. Bogdanova, Natalia N. Antonenkova, Hoda Anton-Culver, Hatef Darabi, Mikael Eriksson, Montserrat Garcia-Closas, Jonine Figueroa, Jolanta Lissowska, Louise Brinton, Peter Devilee, Robert A. E. M. Tollenaar, Caroline Seynaeve, Christi J. van Asperen, Vessela N. Kristensen, kConFab Investigators, Australian Ovarian Cancer Study Group, Susan Slager, Amanda E. Toland, Christine B. Ambrosone, Drakoulis Yannoukakos, Annika Lindblom, Sara Margolin, Paolo Radice, Paolo Peterlongo, Monica Barile, Paolo Mariani, Maartje J. Hooning, John W. M. Martens, J. Margriet Collée, Agnes Jager, Anna Jakubowska, Jan Lubinski, Katarzyna Jaworska-Bieniek, Katarzyna Durda, Graham G. Giles, Catriona McLean, Hiltrud Brauch, Thomas Brüning, Yon-Dschun Ko, The GENICA Network, Hermann Brenner, Aida Karina Dieffenbach, Volker Arndt, Christa Stegmaier, Anthony Swerdlow, Alan Ashworth, Nick Orr, Michael Jones, Jacques Simard, Mark S. Goldberg, France Labrèche, Martine Dumont, Robert Winqvist, Katri Pylkäs, Arja Jukkola-Vuorinen, Mervi Grip, Vesa Kataja, Veli-Matti Kosma, Jaana M. Hartikainen, Arto Mannermaa, Ute Hamann, Georgia Chenevix-Trench, Carl Blomqvist, Kristiina Aittomäki, Douglas F. Easton, Heli Nevanlinna

**Affiliations:** 1 Department of Obstetrics and Gynecology, University of Helsinki and Helsinki University Central Hospital, Helsinki, Finland; 2 Finnish Institute of Occupational Health, Helsinki, Finland; 3 Centre for Cancer Genetic Epidemiology, Department of Public Health and Primary Care, University of Cambridge, Cambridge, United Kingdom; 4 Cancer Epidemiology Centre, Cancer Council Victoria, Melbourne, Australia; 5 Centre for Epidemiology and Biostatistics, Melbourne School of Population and Global Health, The University of Melbourne, Melbourne, Australia; 6 Department of Clinical Genetics, University of Helsinki and Helsinki University Central Hospital, Helsinki, Finland; 7 Department of Oncology, University of Helsinki and Helsinki University Central Hospital, Helsinki, Finland; 8 Human Genetics Division, Genome Institute of Singapore, Singapore, Singapore; 9 Department of Medical Epidemiology and Biostatistics, Karolinska Institutet, Stockholm, Sweden; 10 Division of Cancer Epidemiology, German Cancer Research Center (DKFZ), Heidelberg, Germany; 11 PMV Research Group at the Department of Child and Adolescent Psychiatry and Psychotherapy, University of Cologne, Cologne, Germany; 12 Department of Cancer Epidemiology/Clinical Cancer Registry and Institute for Medical Biometrics and Epidemiology, University Clinic Hamburg-Eppendorf, Hamburg, Germany; 13 Breakthrough Breast Cancer Research Centre, The Institute of Cancer Research, London, United Kingdom; 14 Department of Non-Communicable Disease Epidemiology Department, London School of Hygiene and Tropical Medicine, London, United Kingdom; 15 Centre for Epidemiology and Biostatistics, Melbourne School of Population and Global Health, The University of Melbourne, Melbourne, Australia; 16 Department of Pathology, The University of Melbourne, Melbourne, Australia; 17 Department of Clinical Genetics, VU University Medical Center, Amsterdam, The Netherlands; 18 Department of Medical Genetics, University Medical Center Utrecht, Utrecht, The Netherlands; 19 Division of Gynaecology and Obstetrics, Technische Universität München, Munich, Germany; 20 Division of Molecular Gyneco-Oncology, Department of Gynaecology and Obstetrics, University Hospital of Cologne, Cologne, Germany; 21 Center of Familial Breast and Ovarian Cancer, University Hospital of Cologne, Cologne, Germany; 22 Center for Integrated Oncology (CIO), University Hospital of Cologne, Cologne, Germany; 23 Center for Molecular Medicine Cologne (CMMC), University of Cologne, Cologne, Germany; 24 Max Planck Institute of Psychiatry, Munich, Germany; 25 Institute of Human Genetics, Helmholtz Zentrum München, German Research Center for Environmental Health, Neuherberg, Germany; 26 Section of Cancer Genetics, Institute of Cancer Research, Sutton, United Kingdom; 27 Division of Cancer Epidemiology and Genetics, National Cancer Institute, Rockville, Maryland, United States of America; 28 Program in Molecular and Genetic Epidemiology, Harvard School of Public Health, Boston, Massachusetts, United States of America; 29 Department of Epidemiology, Harvard School of Public Health, Boston, Massachusetts, United States of America; 30 CRUK/YCR Sheffield Cancer Research Centre, Department of Oncology, University of Sheffield, Sheffield, United Kingdom; 31 Academic Unit of Pathology, Department of Neuroscience, University of Sheffield, Sheffield, United Kingdom; 32 Netherlands Cancer Institute, Antoni van Leeuwenhoek hospital, Amsterdam, The Netherlands; 33 University Breast Center Franconia, Department of Gynecology and Obstetrics, University Hospital Erlangen, Friedrich-Alexander University Erlangen-Nuremberg, Comprehensive Cancer Cancer Erlangen-EMN, Erlangen, Germany; 34 David Geffen School of Medicine, Department of Medicine Division of Hematology and Oncology, University of California Los Angeles, California, United States of America; 35 Institute of Human Genetics, University Hospital Erlangen, Friedrich-Alexander University Erlangen-Nuremberg, Comprehensive Cancer Center Erlangen-EMN, Erlangen, Germany; 36 Copenhagen General Population Study, Herlev Hospital, Copenhagen University Hospital, Copenhagen, Denmark; 37 Department of Clinical Biochemistry, Herlev Hospital, Copenhagen University Hospital, Copenhagen, Denmark; 38 Department of Breast Surgery, Herlev Hospital, Copenhagen University Hospital, Copenhagen, Denmark; 39 Human Genetics Group, Human Cancer Genetics Program, Spanish National Cancer Research Centre (CNIO), Madrid, Spain; 40 Centro de Investigación en Red de Enfermedades Raras (CIBERER), Valencia, Spain; 41 Servicio de Oncología Médica, Hospital Universitario La Paz, Madrid, Spain; 42 Servicio de Cirugía General y Especialidades, Hospital Monte Naranco, Oviedo, Spain; 43 Department of Preventive Medicine, Keck School of Medicine, University of Southern California, Los Angeles, California, United States of America; 44 Epidemiology Program, Cancer Research Center, University of Hawaii, Honolulu, Hawaii, United States of America; 45 Centre for Cancer Genetic Epidemiology, Department of Oncology, University of Cambridge, Cambridge, United Kingdom; 46 Clinical Gerontology, Department of Public Health and Primary Care, University of Cambridge, Cambridge, United Kingdom; 47 Department of Laboratory Medicine and Pathology, Mayo Clinic, Rochester, Minnesota, United States of America; 48 Department of Health Sciences Research, Mayo Clinic, Rochester, Minnesota, United States of America; 49 Vesalius Research Center (VRC), VIB, Leuven, Belgium; 50 Laboratory for Translational Genetics, Department of Oncology, University of Leuven, Leuven, Belgium; 51 Oncology Department, University Hospital Gasthuisberg, Leuven, Belgium; 52 Inserm (National Institute of Health and Medical Research), CESP (Center for Research in Epidemiology and Population Health), U1018, Environmental Epidemiology of Cancer, Villejuif, France; 53 University Paris-Sud, UMRS 1018, Villejuif, France; 54 Université Paris Sorbonne Cité, UMR-S775 Inserm, Paris, France; 55 Department of Obstetrics and Gynecology, University of Heidelberg, Heidelberg, Germany; 56 National Center for Tumor Diseases, University of Heidelberg, Heidelberg, Germany; 57 Molecular Epidemiology Group, German Cancer Research Center (DKFZ), Heidelberg, Germany; 58 Research Oncology, Division of Cancer Studies, King's College London, Guy's Hospital, London, United Kingdom; 59 Wellcome Trust Centre for Human Genetics and Oxford Biomedical Research Centre, University of Oxford, Oxford, United Kingdom; 60 Clinical Science Institute, University Hospital Galway, Galway, Ireland; 61 Lunenfeld-Tanenbaum Research Institute of Mount Sinai Hospital, Toronto, Ontario, Canada; 62 Department of Molecular Genetics, University of Toronto, Toronto, Ontario, Canada; 63 Prosserman Centre for Health Research, Lunenfeld-Tanenbaum Research Institute, Mount Sinai Hospital, Toronto, Ontario, Canada; 64 Division of Epidemiology, Dalla Lana School of Public Health, University of Toronto, Toronto, Ontario, Canada; 65 Department of Laboratory Medicine and Pathobiology, University of Toronto, Toronto, Ontario, Canada; 66 Department of Laboratory Medicine, and the Keenan Research Centre of the Li Ka Shing Knowledge Institute, St Michael's Hospital, Toronto, Ontario, Canada; 67 Department of Obstetrics and Gynaecology, Hannover Medical School, Hannover, Germany; 68 Department of Radiation Oncology, Hannover Medical School, Hannover, Germany; 69 N.N. Alexandrov Research Institute of Oncology and Medical Radiology, Minsk, Belarus; 70 Department of Epidemiology, University of California Irvine, Irvine, California, United States of America; 71 Division of Genetics and Epidemiology, Institute of Cancer Research, Sutton, United Kingdom; 72 Breakthrough Breast Cancer Research Centre, Division of Breast Cancer Research, The Institute of Cancer Research, London, United Kingdom; 73 Department of Cancer Epidemiology and Prevention, M. Sklodowska-Curie Memorial Cancer Center and Institute of Oncology, Warsaw, Poland; 74 Department of Human Genetics & Department of Pathology, Leiden University Medical Center, Leiden, Netherlands; 75 Department of Surgical Oncology, Leiden University Medical Center, Leiden, Netherlands; 76 Family Cancer Clinic, Department of Medical Oncology, Erasmus MC-Daniel den Hoed Cancer Center, Rotterdam, Netherlands; 77 Department of Clinical Genetics, Leiden University Medical Center, Leiden, Netherlands; 78 Institute of Clinical Medicine, Faculty of Medicine, University of Oslo, Oslo, Norway; 79 Department of Clinical Molecular Biology (EpiGen), University of Oslo, Oslo, Norway; 80 Department of Genetics, Institute for Cancer Research, Oslo University Hospital, Radiumhospitalet, Oslo, Norway; 81 Peter MacCallum Cancer Center, Melbourne, Australia; 82 QIMR Berghofer Medical Research Institute, Brisbane, Australia; 83 Department of Molecular Virology, Immunology and Medical Genetics, Comprehensive Cancer Center, The Ohio State University, Columbus, Ohio, United States of America; 84 Roswell Park Cancer Institute, Buffalo, New York, United States of America; 85 Molecular Diagnostics Laboratory, IRRP, National Centre for Scientific Research "Demokritos", Aghia Paraskevi Attikis, Athens, Greece; 86 Department of Molecular Medicine and Surgery, Karolinska Institutet, Stockholm, Sweden; 87 Department of Oncology - Pathology, Karolinska Institutet, Stockholm, Sweden; 88 Unit of Molecular Bases of Genetic Risk and Genetic Testing, Department of Preventive and Predictive Medicine, Fondazione IRCCS Istituto Nazionale dei Tumori (INT), Milan, Italy; 89 IFOM, Fondazione Istituto FIRC di Oncologia Molecolare, Milan, Italy; 90 Division of Cancer Prevention and Genetics, Istituto Europeo di Oncologia (IEO), Milan, Italy; 91 Cogentech Cancer Genetic Test Laboratory, Milan, Italy; 92 Department of Medical Oncology, Erasmus University Medical Center, Rotterdam, The Netherlands; 93 Department of Clinical Genetics, Erasmus University Medical Center, Rotterdam, The Netherlands; 94 Department of Genetics and Pathology, Pomeranian Medical University, Szczecin, Poland; 95 Postgraduate School of Molecular Medicine, Warsaw Medical University, Warsaw, Poland; 96 Anatomical Pathology, The Alfred Hospital, Melbourne, Australia; 97 Dr. Margarete Fischer-Bosch-Institute of Clinical Pharmacology, Stuttgart, Germany; 98 University of Tübingen, Tübingen, Germany; 99 Institute for Prevention and Occupational Medicine of the German Social Accident Insurance, Institute of the Ruhr-University Bochum (IPA), Bochum, Germany; 100 Department of Internal Medicine, Evangelische Kliniken Bonn gGmbH, Johanniter Krankenhaus, Bonn, Germany; 101 Molecular Genetics of Breast Cancer, German Cancer Research Center (DKFZ), Heidelberg, Germany; 102 Institute for Occupational Medicine and Maritime Medicine, University Medical Center Hamburg-Eppendorf, Hamburg, Germany; 103 Institute of Pathology, Medical Faculty of the University of Bonn, Bonn, Germany; 104 Division of Clinical Epidemiology and Aging Research, German Cancer Research Center (DKFZ), Heidelberg, Germany; 105 German Cancer Consortium (DKTK), Heidelberg, Germany; 106 Saarland Cancer Registry, Saarbrücken, Germany; 107 Division of Genetics and Epidemiology and Division of Breast Cancer Research, The Institute of Cancer Research, Sutton, Surrey, United Kingdom; 108 Cancer Genomics Laboratory, Centre Hospitalier Universitaire de Québec Research Center and Laval University, Quebec, Canada; 109 Department of Medicine, McGill University, Montreal, Canada; 110 Division of Clinical Epidemiology, McGill University Health Centre, Royal Victoria Hospital, Montreal, Quebec, Canada; 111 Départements de Santé Environnementale et Santé au Travail et de Médecine Sociale et Préventive, Université de Montréal, Montreal, Quebec, Canada; 112 Laboratory of Cancer Genetics and Tumor Biology, Department of Clinical Chemistry and Biocenter Oulu, University of Oulu, NordLab Oulu/Oulu University Hospital, Oulu, Finland; 113 Department of Oncology, Oulu University Hospital, University of Oulu, Oulu, Finland; 114 Department of Surgery, Oulu University Hospital, University of Oulu, Oulu, Finland; 115 School of Medicine, Institute of Clinical Medicine, Oncology, University of Eastern Finland, Kuopio, Finland; 116 Cancer Center, Kuopio University Hospital, Kuopio, Finland; 117 School of Medicine, Institute of Clinical Medicine, Pathology and Forensic Medicine, University of Eastern Finland, Kuopio, Finland; 118 Imaging Center, Department of Clinical Pathology, Kuopio University Hospital, Kuopio, Finland; 119 Cancer Center of Eastern Finland, University of Eastern Finland, Kuopio, Finland; 120 Department of Genetics, QIMR Berghofer Medical Research Institute, Brisbane, Australia; Vanderbilt University Medical Center, United States of America

## Abstract

Genetic variations, such as single nucleotide polymorphisms (SNPs) in microRNAs (miRNA) or in the miRNA binding sites may affect the miRNA dependent gene expression regulation, which has been implicated in various cancers, including breast cancer, and may alter individual susceptibility to cancer. We investigated associations between miRNA related SNPs and breast cancer risk. First we evaluated 2,196 SNPs in a case-control study combining nine genome wide association studies (GWAS). Second, we further investigated 42 SNPs with suggestive evidence for association using 41,785 cases and 41,880 controls from 41 studies included in the Breast Cancer Association Consortium (BCAC). Combining the GWAS and BCAC data within a meta-analysis, we estimated main effects on breast cancer risk as well as risks for estrogen receptor (ER) and age defined subgroups. Five miRNA binding site SNPs associated significantly with breast cancer risk: rs1045494 (odds ratio (OR) 0.92; 95% confidence interval (CI): 0.88–0.96), rs1052532 (OR 0.97; 95% CI: 0.95–0.99), rs10719 (OR 0.97; 95% CI: 0.94–0.99), rs4687554 (OR 0.97; 95% CI: 0.95–0.99, and rs3134615 (OR 1.03; 95% CI: 1.01–1.05) located in the 3′ UTR of *CASP8*, *HDDC3*, *DROSHA*, *MUSTN1*, and *MYCL1*, respectively. *DROSHA* belongs to miRNA machinery genes and has a central role in initial miRNA processing. The remaining genes are involved in different molecular functions, including apoptosis and gene expression regulation. Further studies are warranted to elucidate whether the miRNA binding site SNPs are the causative variants for the observed risk effects.

## Introduction

Breast cancer is the most common women's cancer and is a leading cause of cancer mortality [1]. Inherited genetic variation has been associated with the initiation, development and progression of breast cancer. Studies on twins have suggested that hereditary predisposing factors are involved in up to one third of all breast cancers [2]. Many genetic loci have been associated with breast cancer risk and collectively explain approximately 35% of the familial risk [3], [4]. The largest genetic association study of breast cancer to date identified 41 novel low penetrance susceptibility loci [4] by selecting nearly 30,000 SNPs from a meta-analysis of nine genome-wide association (GWA) studies and genotyping them using 41,785 cases and 41,880 controls of European ancestry from studies in the Breast Cancer Association Consortium (BCAC). These 41 susceptibility loci probably represent the tip of the ice berg, and additional SNPs from the combined GWAS might explain a similar fraction of familial risk to that attributed to the already identified loci [4].

Mature miRNAs are 20–23 nucleotide, single-stranded RNA molecules that play a crucial role in gene expression regulation for many cellular processes including differentiation potential and development pattern. MiRNAs undergo a stepwise maturation process involving an array of miRNA machinery components. Drosha and DGCR8 mediate the cleavage of long primary miRNA transcripts (pri-miRNAs) into shorter pre-miRNAs in the nucleus [5], [6]. The pre-miRNAs are then transported to the cytoplasm where they are further cleaved by Dicer to produce mature miRNAs [7]. MiRNAs interact by pairing with the 3′ untranslated region (UTR), and also within the coding region and 5′ UTR of the corresponding mRNAs leading to mRNA destabilization, cleavage or translation repression. More effective mRNA destabilization is achieved when miRNA targets the 3'UTR rather than other mRNA regions [8]–[10]. An individual miRNA may regulate approximately 100 distinct mRNAs, and together more than 1000 human miRNAs are believed to modulate more than half of the mRNA species encoded in the genome [11], [12]. Additionally, most mRNAs possess binding sites for miRNAs [13]. MiRNAs are involved in tumorigenesis in that they can be either oncogenic when tumor suppressor genes are targeted, or genomic guardians (tumour suppressor miRNAs) when oncogenes are targeted [14]. Additionally it has been suggested that they may modulate both metastasis [15] and chemotherapy resistance [16]. MiRNAs have also been shown to have altered expression levels in tumours compared to normal tissue and between tumor subtypes in breast cancer among other carcinoma types [17]–[19]. SNPs may affect miRNA machinery genes or miRNAs activity; however SNPs can also create, abolish or modify miRNA binding sites in their binding regions. Polymorphisms in miRNA binding sites have been studied in regard to the risk of several cancers [20], including breast cancer [21]–[23]. These studies have found evidence for association of miRNA related SNPs and cancer risk, but the study sample sizes have been relatively small.

In this study, we investigate associations between miRNA-related polymorphisms and breast cancer risk by using a meta-analysis of nine GWAS and subsequent genotyping of top hits using 41,785 cases and 41,880 controls of European ancestry from the BCAC. To our knowledge, this is thus far the largest investigation of associations between miRNA-related polymorphisms and breast cancer susceptibility.

## Materials and Methods

### SNP selection and genotyping

SNPs in mature or pre-miRNAs, in genes of the miRNA machinery and in 3'UTR regions of protein coding genes with a potential effect on miRNA binding were systematically searched from Ensembl (hg18/build36) and Patrocles databases [24]. Additionally, tagging SNPs for such with r^2^≥0.8 were also identified utilizing the public HapMap SNP database. By this *in silico* approach we identified altogether 147,801 candidate SNPs and 12,550 tagging SNPs. These SNPs were then overlayed with those from the combined GWAS from the BCAC [4] and altogether 2196 SNPs were present (either genotyped or imputed) in the combined GWAS. These SNPs were genotyped with Illumina or Affymetrix arrays, as described previously [25]–[32]. The combined GWAS data were imputed for all scans using HapMap version 2 CEU as a reference in similar fashion to that presented by Michailidou and colleagues [4] with the exception that the HapMap version 2 release 21 was used at the time the overlay was performed. Analysis using a 1-degree-of-freedom trend test of these 2196 SNPs in the combined GWAS indicated some evidence of association with breast cancer risk for 44 SNPs (p<0.09). Notably, the combined GWAS included imputed data generated using HapMap version 2 release 21 (based on NCBI build 35 (dbSNP b125)), whereas the results presented here for the combined GWAS are based on imputation using HapMap version 2 release 22 (based on NCBI build 36 (dbSNP b126)). In the release 22, a number of SNPs were excluded due to mapping inconsistencies in build 35 relative to build 36. Hence, the estimates from the combined GWAS may slightly differ from the initial association analysis. The 44 SNPs (including 30 candidate and 14 tagging SNP) were genotyped on additional samples in the BCAC using the custom Illumina Infinium array (iCOGS) which included a total of 211,155 SNPs as described previously. The detailed description of quality control process for combined GWAS and iCOGS genotyping data was presented in [4].

**Figure 1:**
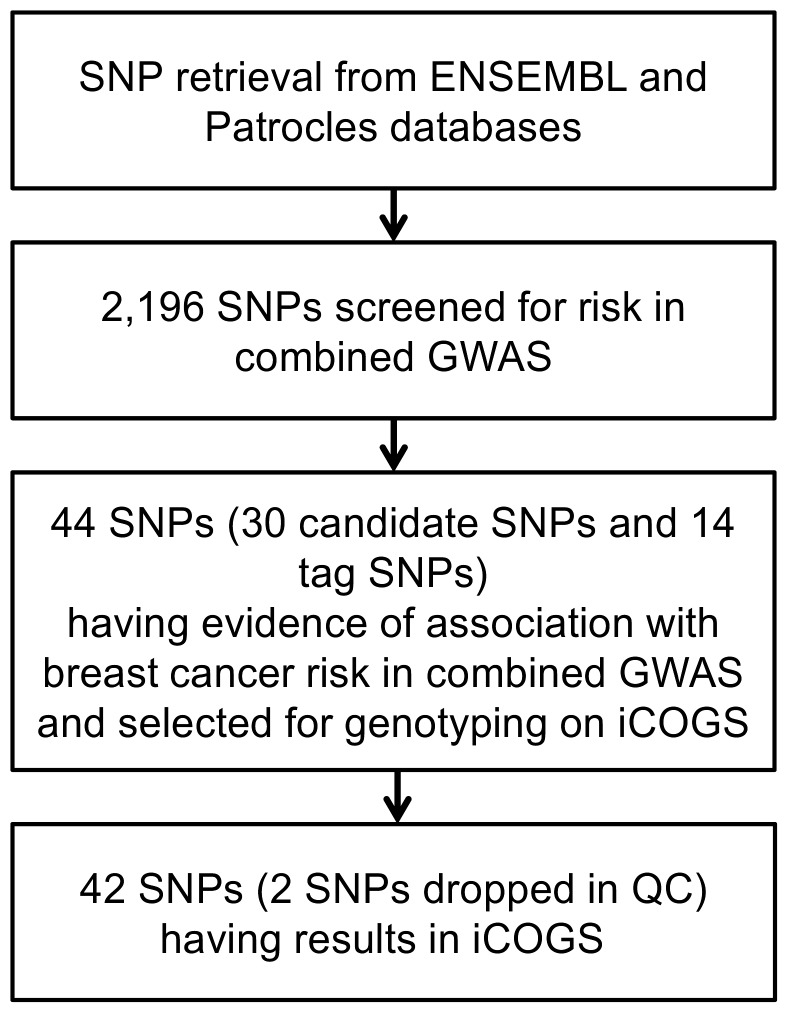
Workflow of miRNA SNP selection.

Of the 42 SNPs that passed quality control [4], two were located in miRNA genes (one candidate SNP located in pre-miRNA hsa-miR-2110 and one tag SNP tagging a mature hsa-mir-548l variant), and four SNPs were located in miRNA machinery genes (*SMAD5*, *SND1*, *CNOT4* and *DROSHA*). The genotyped *DROSHA* SNP tags the 3′ UTR miRNA binding site variant in the DROSHA gene. The remaining 38 candidate or tag SNPs were located in, or tagged to a predicted miRNA binding site in the 3′ UTR of protein coding genes. All 42 SNPs are described in Table 1. The workflow of the SNP selection in different stages is illustrated in Figure 1.

**Table 1:** The 42 studied SNPs in miRNAs, miRNA machinery genes and miRNA target genes.

Functional SNP (Tag SNP, R-squared)	Chr	Position	Coding	Gene		miRNA		SNP effect^a^
Located within miRNA								
rs17091403	10	115923895	GA		hsa-miR-2110			
rs13447640 (rs1805360, r^2^ = 1)	11	93866677	GA		hsa-mir-548l			
Located in miRNA biogenesis machinery genes								
rs3764941	5	135497426	AC		SMAD5			
rs17151639	7	127425052	AG		SND1			
rs17480616	7	134773600	CG		CNOT4			
rs10719	5	31437204	GA		DROSHA		hsa-miR-1298	AC
Located in miRNA target genes								
rs2550303	16	54953111	AG		AMFR		hsa-miR-577	AC
rs7513934	1	52590776	GA		CC2D1B		hsa-miR-384/hsa-miR-577	CNC
rs1128226	7	21908194	AC		CDCA7L		hsa-miR-548g	AC
rs3796133	3	100000533	GA		DCBLD2		hsa-miR-624*	AC
rs7441	12	90063806	GA		DCN		hsa-miR-135b*	AC
rs1803439	21	37807312	AG		DYRK1A		hsa-miR-550	AC
rs3797	15	27199858	AG		FAM189A1		hsa-miR-570	AC
rs7130622	11	128186721	AC		FLI1		hsa-miR-138-2*	AC
rs1052532	15	89275240	AG		HDDC3		hsa-miR-1224-3p/hsa-miR-1260/hsa-miR-1280	AC
rs7040123	9	7160742	AG		KDM4C		hsa-miR-154*/hsa-miR-487a	AC
rs1062225	10	49313232	AG		MAPK8		hsa-miR-203	AC
rs41739	7	116224740	AG		MET		hsa-miR-576-5p	AC
rs702681	5	56253786	AG		MIER3		hsa-miR-196a*	AC
rs3134615	1	40134653	CA		MYCL1		hsa-miR-1827	ANC
rs2304669	2	238830402	AG		PER2		hsa-miR-885-3p	AC
rs13422	17	15074900	AC		PMP22		hsa-miR-29b-1*	AC
rs7562391	2	201444411	AC		PPIL3		hsa-miR-493*/hsa-miR-499-3p	AC
rs7520333	1	40862837	AG		RIMS3		hsa-let-7d/hsa-let-7e	CNC
rs739692	18	53178524	GA		ST8SIA3		hsa-miR-96/hsa-miR-1271/hsa-miR-182	AC
rs1058450	4	120200088	GA		SYNPO2		hsa-miR-183	AC
rs4351800	11	7446395	CA		SYT9		hsa-miR-544	AC
rs12438324	15	55366808	AG		TCF12		hsa-miR-591	AC
rs12869870	13	99415306	GA		ZIC5		hsa-miR-34a/hsa-miR-34c-5p/hsa-miR-449a/hsa-miR-449b	AC
rs9990 (rs1444418, r^2^ = 1)	10	64230476	AG		ADO		hsa-miR-512-5p/hsa-miR-510	AC
rs757537 (rs4705870, r^2^ = 1)	5	132187033	GA		ANKRD43		hsa-miR-320a/hsa-miR-320b/hsa-miR-320c/hsa-miR-320d	AC
rs3774729 (rs2037119, r^2^ = 0.943)	3	63969919	GA		ATXN7		hsa-miR-1206	AC
rs1045487 (rs1045494, r^2^ = 1)	2	201860026	AG		CASP8		hsa-miR-938	AC
rs7288826 (rs8140217, r^2^ 1)	22	37547947	GA		CBX6		hsa-miR-1207-5p	AC
rs17569034 (rs17512204, r^2^ = 0.835)	2	118449301	GA		CCDC93		hsa-miR-1178	AC
rs3205281 (rs7674744, r^2^ = 1)	4	78874296	GA		CNOT6L		hsa-miR-643/hsa-miR-297	AC
rs13005 (rs9473, r^2^ = 0.964)	10	13727177	GA		FRMD4A		hsa-miR-548m	AC
rs3809831 (rs3809828, r^2^ = 1)	17	7187575	GA		KCTD11		hsa-miR-892b	AC
rs6445538 (rs4687554, r^2^ = 1)	3	52839175	AG		MUSTN1		hsa-miR-891b	AC
rs7818 (rs9371201, r^2^ = 0.875)	6	150186694	GA		PCMT1		hsa-miR-595	AC
rs9844202 (rs7635553, r^2^ = 1)	3	168646064	GA		SERPINI2		hsa-miR-1272	AC
rs2271565 (rs7086917, r^2^ = 1)	10	49867441	AC		WDFY4		hsa-miR-657/hsa-miR-214/hsa-miR-15a/hsa-miR-16/hsa-miR-15b/hsa-miR-195/hsa-miR-424/hsa-miR-497	AC

Tag SNPs used in the analysis are presented in the parenthesis along with the R squred value relative to the functional SNP.

a According to Patrocles prediction; AC  =  abolishes conserved binding site, ANC  =  abolishes non-conserved binding site, CNC  =  creates non-conserved binding site (Target sites are considered conserved if they are shared by at least one primate, one rodent and one nonprimate/nonrodent mammal [24]).

### Study sample

The combined GWAS included nine breast cancer studies totalling 10,052 cases and 12,575 controls of European ethnic background. Details and study-specific subject numbers are presented in Table S1. Since the GWAS were limited to patients of European ethnic background we further utilized 41,785 cases ascertained for their first primary, invasive breast cancer and 41,880 controls of European ancestry from 41 BCAC studies genotyped using the iCOGS array (Table S2). For a subgroup analysis of ER negative and ER positive cases, as well as cases aged less than 50 years at diagnosis, we included all the cases for which the respective data were available. The ER subgroup analysis was based on 702 ER negative cases and 2,019 ER positive cases from five GWAS studies and 7,200 ER negative cases from 40 BCAC studies and 26,302 ER positive cases from 34 BCAC studies. The analysis of cases aged less than 50 years at diagnosis was based on 3,470 cases from three GWAS studies and 9,483 cases from 35 BCAC studies. All participating studies conform to the Declaration of Helsinki and were approved by the respective ethical review boards and ethics committees (Tables S1 and S2), and all participants in these studies had provided written consent for the research.

### Statistical methods

We used logistic regression to estimate per-allele log-odds ratios and standard errors including the study as a covariate. We also included principal components as covariates in order to correct for potential hidden population structure. In the GWAS, for two studies (UK2 and HEBCS) the estimates were adjusted for the first three principal components and in the iCOGS analysis we used the first six principal components and an additional component to reduce inflation for the LMBC study, as described previously [4]. Subgroup analyses were carried out for ER negative and positive subgroups and for the group aged less than 50 years at diagnosis. For meta-analysis, we combined the estimates from the combined GWAS and iCOGS with a fixed effects model using the inverse variance weighted method. In the meta-analysis, the subjects involved in both combined GWAS and iCOGS (1880) were only taken into account once. In order to adust for *P*-values against multiple testing, we used Benjamini Hochberg correction. The adjusted *P*-values are shown in Table 2 along with the nominal *P*-values. In the text we report the nominal *P*-values. The statistical analyses were conducted using the R 2.14.0 statistical computing environment (http://www.r-project.org/).

**Table 2:** Associations of SNPs in the GWAS and iCOGS separately and combined GWAS + iCOGS and breast cancer risk.

SNP	Chr	Position	coding^1^	GWAS OR (95%CI)^2^	GWAS *P* 3	iCOGS OR (95% CI)^2^	iCOGS *P* 3	Combined GWAS + iCOGS OR (95% CI)^2^	Combined GWAS + iCOGS *P* 3(BH corrected *P*)^4^	Gene
rs702681	5	56253786	AG	1.07 (1.02–1.11)	3.92×10^−3^	1.06 (1.04–1.09)	2.76×10^−8^	1.06 (1.04–1.08)	3.88×10^−10^ (1.63×10^−8^)	MIER3
rs1045494	2	201860026	AG	0.90 (0.81–1.00)	4.74×10^−2^	0.92 (0.88–0.96)	4.47×10^−4^	0.92 (0.88–0.96)	5.94×10^−5^ (1.25×10^−3^)	CASP8
rs1052532	15	89275240	AG	0.94 (0.90–0.98)	7.94×10^−3^	0.97 (0.95–0.99)	1.47×10^−2^	0.97 (0.95–0.99)	7.78×10^−4^ (1.09×10^−2^)	HDDC3
rs10719	5	31437204	GA	0.92 (0.88–0.97)	8.79×10^−4^	0.98 (0.95–1.00)	5.32×10^−2^	0.97 (0.94–0.99)	1.35×10^−3^ (1.42×10^−2^)	DROSHA
rs4687554	3	52839175	AG	0.94 (0.90–0.99)	1.23×10^−2^	0.97 (0.95–1.00)	2.39×10^−2^	0.97 (0.95–0.99)	1.71×10^−3^ (1.44×10^−2^)	MUSTN1
rs3134615	1	40134653	CA	1.04 (0.99–1.09)	9.97×10^−2^	1.03 (1.00–1.05)	2.09×10^−2^	1.03 (1.01–1.05)	5.07×10^−3^ (3.55×10^−2^)	MYCL1
rs7635553	3	168646064	GA	0.89 (0.83–0.95)	9.73×10^−4^	0.98 (0.95–1.01)	1.98×10^−1^	1.00 (0.97–1.04)	9.24×10^−3^ (5.54×10^−2^)	SERPINI2
rs3796133	3	100000533	GA	1.18 (1.08–1.29)	4.18×10^−4^	1.01 (0.97–1.06)	5.74×10^−1^	1.04 (1.00–1.09)	3.93×10^−2^ (1.45×10^−1^)	DCBLD2
rs4351800	11	7446395	CA	1.04 (1.00–1.08)	4.48×10^−2^	1.01 (0.99–1.03)	1.98×10^−1^	1.02 (1.00–1.04)	4.15×10^−2^ (1.45×10^−1^)	SYT9
rs17512204	2	118449301	GA	1.06 (0.98–1.14)	1.20×10^−1^	1.03 (0.99–1.06)	1.63×10^−1^	1.03 (1.00–1.07)	5.22×10^−2^ (1.57×10^−1^)	CCDC93
rs3809828	17	7187575	GA	1.17 (1.06–1.28)	1.97×10^−3^	1.01 (0.97–1.05)	5.22×10^−1^	0.99 (0.95–1.03)	7.93×10^−2^ (2.22×10^−1^)	KCTD11
rs7441	12	90063806	GA	1.11 (1.03–1.20)	8.70×10^−3^	1.01 (0.97–1.05)	5.98×10^−1^	1.03 (0.99–1.06)	1.04×10^−1^ (2.57×10^−1^)	DCN
rs7086917	10	49867441	AC	0.96 (0.93–1.00)	6.35×10^−2^	0.99 (0.97–1.01)	4.38×10^−1^	0.99 (0.97–1.00)	1.29×10^−1^ (3.01×10^−1^)	WDFY4
rs7040123	9	7160742	AG	1.11 (0.99–1.23)	7.59×10^−2^	1.02 (0.97–1.07)	5.14×10^−1^	1.00 (0.95–1.04)	1.79×10^−1^ (3.74×10^−1^)	KDM4C
rs7674744	4	78874296	GA	0.94 (0.89–0.99)	2.83×10^−2^	0.99 (0.97–1.02)	6.91×10^−1^	1.01 (0.98–1.03)	1.81×10^−1^ (3.74×10^−1^)	CNOT6L
rs12438324	15	55366808	AG	0.87 (0.79–0.97)	1.01×10^−2^	1.00 (0.94–1.05)	8.69×10^−1^	1.02 (0.98–1.07)	1.87×10^−1^ (3.74×10^−1^)	TCF12
rs17151639	7	127425052	AG	0.96 (0.92–1.01)	1.09×10^−1^	0.99 (0.97–1.02)	5.66×10^−1^	1.00 (0.98–1.02)	2.19×10^−1^ (4.18×10^−1^)	SND1
rs17480616	7	134773600	CG	0.87 (0.72–1.04)	1.27×10^−1^	0.99 (0.93–1.04)	6.39×10^−1^	0.98 (0.92–1.03)	3.70×10^−1^ (5.98×10^−1^)	CNOT4
rs7513934	1	52590776	GA	1.04 (1.00–1.08)	7.98×10^−2^	1.00 (0.98–1.02)	9.99×10^−1^	1.01 (0.99–1.02)	4.37×10^−1^ (6.34×10^−1^)	CC2D1B
rs2304669	2	238830402	AG	0.96 (0.91–1.02)	1.86×10^−1^	1.00 (0.97–1.02)	8.17×10^−1^	0.99 (0.97–1.02)	4.38×10^−1^ (6.34×10^−1^)	PER2
rs1058450	4	120200088	GA	0.96 (0.91–1.01)	1.33×10^−1^	1.00 (0.97–1.02)	9.28×10^−1^	1.01 (0.98–1.03)	4.59×10^−1^ (6.43×10^−1^)	SYNPO2

The SNPs with consistent odds ratios in combined GWAS and iCOGS analysis are shown. (Results for all 42 SNPs are presented in Table S3.)

1 Build 36 position.

2 Per allele odds ratio for the minor allele relative to the major allele.

3 1df p-trend.

4 1df p-trend adjusted against multiple testing by Benjamini–Hochberg correction method.

## Results

For the 42 SNPs we successfully genotyped, estimates of association from the combined GWAS and from iCOGS analysis are shown in Table S3. Twenty-one SNPs showed consistent associations with breast cancer risk in the combined GWAS and in iCOGS analysis; results from the meta-analysis are shown in Table 2. The most significantly associated SNP, rs702681 (OR 1.06 [95%CI 1.04–1.08]; *P* 3.9×10^−10^), is located in the 3'UTR of MIER3, close to the known breast cancer susceptibility gene MAP3K1. The SNP rs702681 is located at the same 5q11.2 locus as the previously published risk SNP rs889312 [33] (correlation r^2^ = 0.3). When the two SNPs were analysed in the same logistic regression model, the association with rs889312, but not that with rs702681 remained nominally statistically significant, suggesting that rs702681 is unlikely to be the causal SNP at this locus. The five SNPs with the significant novel associations from the meta-analysis (*P*≤5.07×10^−3^and adjusted *P*≤3.55×10^−2^ after correction for multiple testing) were rs1045494, (OR 0.92 [95%CI 0.88–0.96]; *P* =  5.90×10^−5^), rs1052532, (OR 0.97 [95%CI 0.95–0.99]; *P* = 7.78×10^−4^), rs10719, (OR 0.97 [95%CI 0.94–0.99]; *P* = 1.35×10^−3^) rs4687554 (OR 0.97 [95%CI 0.95–0.99]; *P* = 1.71×10^−3^) and rs3134615 (OR 1.03 [95%CI 1.01–1.05]; *P* = 5.07×10^−3^) located in 3′ UTR of Caspase-8 (*CASP8*), HD Domain Containing 3 (*HDDC3*), *DROSHA*, Musculoskeletal, Embryonic Nuclear Protein 1 (*MUSTN1*) and V-Myc Myelocytomatosis Viral Oncogene Homolog 1 (*MYCL1*), respectively (Table 2). SNP rs1045494 is tagging the hsa-miR-938 binding site SNP rs1045487 (r^2^ = 1.0) of CASP8 and the SNP rs1052532 in *HDDC3* is predicted to abolish the binding site for hsa-miR-1224-3p. The SNP rs10719 is predicted to abolish the hsa-miR-1298 binding site in the 3′ UTR of *DROSHA*. SNP rs4687554 tags the hsa-miR-891b binding site SNP rs6445538 (r^2^ = 1.0) of *MUSTN1* and rs3134615 is located at the binding site of hsa-miR-1827 of *MYCL1*. There was no evidence for heterogeneity in the per-allele OR for any SNP. The per study per allele ORs for these five miRNA binding site SNPs from the combined GWAS along with per-SNP heterogeneity variance *P*-values are shown in Figure S1 and from the iCOGS in Figure S2. Next we analysed the SNPs by ER status-defined subtype, and for cases aged less than 50 years at diagnosis, for risk associations in the meta-analysis of combined GWAS and iCOGS (Tables S4, S5 and S6). These analyses did not reveal any additional significant results. For rs1045494 in *CASP8*, rs4687554 in *MUSTN1* and rs3134615 in *MYCL1* (OR 1.03 [95%CI 1.01–1.05]; *P* = 7.75×10^−4^) a more significant association with breast cancer risk was found for the ER positive subgroup than in the main analysis, but the result from the test for heterogeneity by ER status was not significant (data not shown). All associations were estimated using an additive inheritance model. Dominant and recessive models did not improve the estimates (data not shown).

## Discussion

We investigated associations between genetic variation in miRNAs, in the genes of the miRNA machinery and in the miRNA binding sites and the risk of breast cancer. We identified several SNPs that are predicted to abolish an miRNA binding site and that are significantly associated with breast cancer risk. Previous studies investigating miRNA related SNPs, especially in miRNA binding sites have included predefined sets of genes. Nicoloso and colleagues investigated 38 previously identified breast cancer risk SNPs and found two to modify miRNA binding sites in TGFB1 and XRCC1 in vitro [23]. Neither of these were included in our data set. Liang and colleagues investigated 134 potential miRNA binding sites in cancer-related genes and found six miRNA binding site SNPs that were associated with ovarian cancer risk [34].

In the meta-analysis of combined GWAS and iCOGS for main effects, for four of the five most significant miRNA binding site SNPs, the minor allele was associated with a decreased breast cancer risk. The minor allele of SNP rs3134615 in 3′ UTR of *MYCL1* was associated with an increased breast cancer risk. All the five most significant miRNA binding site SNPs locate in 3′ UTR and have been predicted to abolish the miRNA binding site. The defect in miRNA-mediated regulation would be expected to lead to an increase in the translation of the corresponding encoded protein. The five genes, whose regulation may be affected by the miRNA-associated SNPs, include the pre-apoptotic gene *CASP8*, *HDDC3*, miRNA biogenesis master regulator *DROSHA*, MYC-family member *MYCL1* and *MUSTN1*. *CASP8* is involved in apoptosis in breast cancer cells [35], and many studies have reported polymorphisms in this gene to be associated with risks for several cancers [36], [37] including breast cancer [38], [39], indicating the importance of *CASP8* in tumor development. SNP rs1045494 studied here is located close to the coding region SNP rs1045485 that has been previously shown to have a stronger protective effect [38], [40], [41]. Interestingly, Michalidou and colleagues reported this SNP as having only weak evidence for an association (*P* 0.0013 in combined GWAS and iCOGS) [4], but these two SNPs (rs1045485 and rs1045494) are not correlated (r^2^ = 0.001 in Caucasian population). Neither is rs1045494 correlated with the more strongly associated rs1830298 SNP, identified through fine-mapping of the region (r^2^ = 0.02) [42]. Rs1045494 tags SNP rs1045487 (r^2^ = 1.0) which is predicted to abolish the hsa-miR-938 binding site and thus may affect *CASP8* expression. There is very little reported evidence on the involvement of *HDDC3* or the hsa-miR-1224-3p in cancer, indicating a novel association with risk. *HDDC3* has been suggested to be involved in the starvation response [43]. The *HDDC3* gene is expressed at higher levels by several different tumor types, including breast tumors, than by normal tissue [44]. *DROSHA* is a miRNA master regulator. It is a member of the RNase III enzyme family, belongs to the miRNA biogenesis pathway and is the core nuclease that processes pri-miRNAs into pre-miRNAs in the nucleus [5], [6]. The SNP rs10719 in the 3′ UTR of *DROSHA* is predicted to abolish the hsa-miR-1298 binding site. Hsa-miR-1298 is predicted to target *DROSHA* by the Patrocles prediction as well as by TargetScan [45] and PITA [46] prediction algorithms. Recently a small Korean study reported another SNP rs644236, tagging the SNP rs10719 (r^2^ = 0.955 in CEU population and r^2^ = 0.876 in Asian population (combined CHB and JPT)) to be associated with elevated breast cancer risk [47]. When taking into account the opposite major and minors alleles in the Asian and European populations for SNPs rs644236 and rs10719, this result is in concordance with our results where both the combined GWAS as well as the iCOGS analysis consistently indicated an association of the minor allele of SNP rs10719 with reduced breast cancer risk. We also found the minor allele of SNP rs3134615 in the 3′ UTR of *MYCL1* to be associated with an increased risk. *MYCL1* (L-MYC) belongs to the same family of transcription factors as the known proto-oncogene MYC (*C-MYC*) and they share a high degree of structural similarity [48]. The *MYCL1* gene has previously been reported to be amplified and overexpressed in ovarian cancer [49]. A case-control study by Xiong and colleagues reported SNP rs3134615 to be significantly associated with increased risk of small cell lung cancer [50]. SNP rs3134615 was predicted by Patrocles to abolish the hsa-miR-1827 binding site. This has also been suggested by functional studies where *MYCL1* was found as the target of hsa-miR-1827 and the SNP rs3134615 was also found to increase *MYCL1* expression [50]. The evidence from functional studies is consistent with our finding that SNP rs3134615 might increase breast cancer risk. *MUSTN1* has been shown to be involved in the development and regeneration of the musculoskeletal system [51]. Thus far no evidence of association between *MUSTN1* and breast cancer has been reported, but the *MUSTN1* gene is expressed in the mammary glands [52].

Since only a small fraction of miRNA binding sites has been experimentally validated, we selected SNPs that had been computationally predicted to affect miRNA binding sites. For our original SNP selection we used the Patrocles database that contains predicted miRNA binding sites and also compiles perturbation prediction of SNP effects. There are a multitude of prediction programs and their performance has been evaluated [53]. Witkos and colleagues find target prediction algorithms that utilize orthologous sequence alignment, like Patrocles, to be the most reliable.

The followup of the 42 miRNA related SNPs identified five significant associations with breast cancer risk. Although the individual risk effects were subtle, considering that we could only investigate a small proportion of our initial *in silico* data set of miRNA related SNPs (over 140,000 SNPs) this may suggest that genetic polymorphisms affecting the miRNA regulation could have a considerable combined effect on breast cancer risk.

It should be noted that, until fine mapping studies are carried out for these loci, it is not clear whether these miRNA-related SNPs are the variants responsible for the observed associations.

This comprehensive analysis of miRNA related polymorphisms using a large two stage study of women with European ancestry provides evidence for miRNA related SNPs being potential modulators of breast cancer risk.

## Supporting Information

Figure S1 **Forest plots for the five most significant miRNA binding site SNPs from the combined GWAS.** Squares indicate the estimated per-allele OR for the minor allele in Europeans. The horizontal lines indicate 95% confidence limits. The vertical blue dashed lines indicate clipping of the confidence intervals for presentation purpose. The area of the square is inversely proportional to the variance of the estimate. The diamond indicates the estimated per-allele OR from the combined analysis.
(PDF)

Figure S2 **Forest plots for the five most significant miRNA binding site SNPs from the iCOGS.** Squares indicate the estimated per-allele OR for the minor allele in Europeans. The horizontal lines indicate 95% confidence limits. The vertical blue dashed lines indicate clipping of the confidence intervals for presentation purpose. The area of the square is inversely proportional to the variance of the estimate. The diamond indicates the estimated per-allele OR from the combined analysis.
(PDF)

Table S1 **A description of each GWAS study, number of subjects and genotyping platform used in combined GWAS.**
(DOC)

Table S2 **A description of each BCAC study with subjects of European origin in iCOGS.**
(DOC)

Table S3 **Frequencies and effect sizes of the 42 SNPs in the main analysis; combined GWAS and iCOGS.**
(DOC)

Table S4 **Results for SNPs in the GWAS and iCOGS separately and combined GWAS+iCOGS analysis for ER negative subgroup.**
(DOC)

Table S5 **Results for SNPs in the GWAS and iCOGS separately and combined GWAS+iCOGS analysis for ER positive subgroup.**
(DOC)

Table S6 **Results for SNPs in the GWAS and iCOGS separately and combined GWAS+iCOGS analysis for cases less than 50 years at diagnosis.**
(DOC)
