# Supplementary figures and images for: MicroRNA Related Polymorphisms and Breast Cancer Risk
Source: PLoS One. 2014 Nov 12;9(11):e109973. doi: 10.1371/journal.pone.0109973 (PMC4229095; doi:10.1371/journal.pone.0109973)

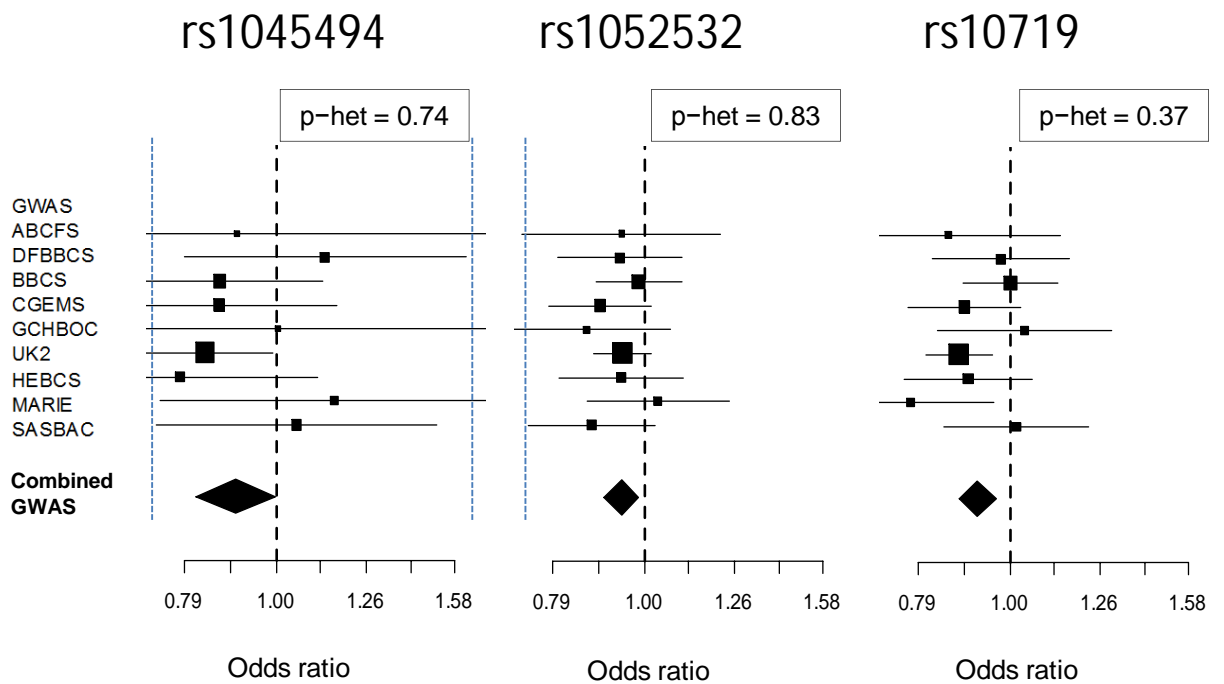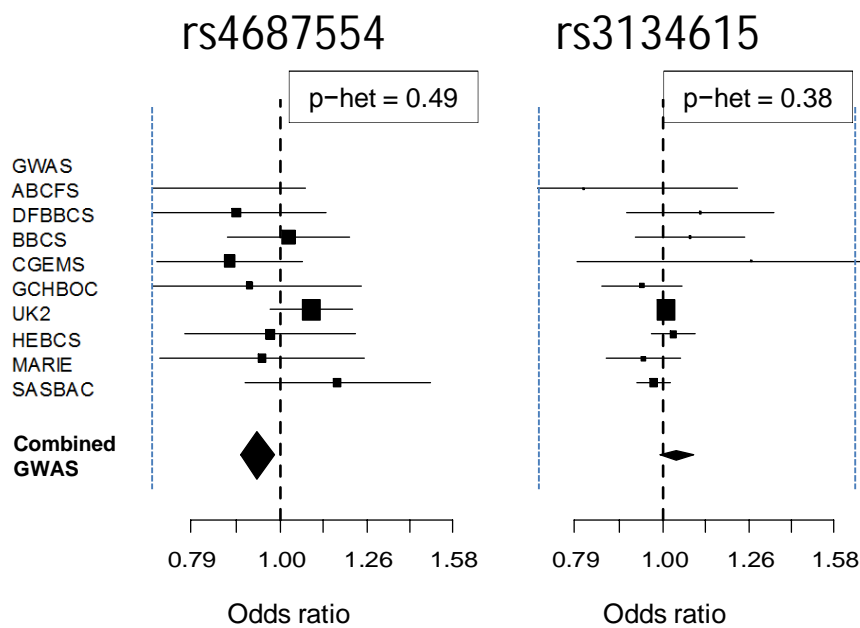

Supplement: Figure S1 — Forest plots for the five most significant miRNA binding site SNPs from the combined GWAS. Squares indicate the estimated per-allele OR for the minor allele in Europeans. The horizontal lines indicate 95% confidence limits. The vertical blue dashed lines indicate clipping of the confidence intervals for presentation purpose. The area of the square is inversely proportional to the variance of the estimate. The diamond indicates the estimated per-allele OR from the combined analysis. (PDF) [file pone.0109973.s001.pdf]

rs1045494

p-het = 0.22

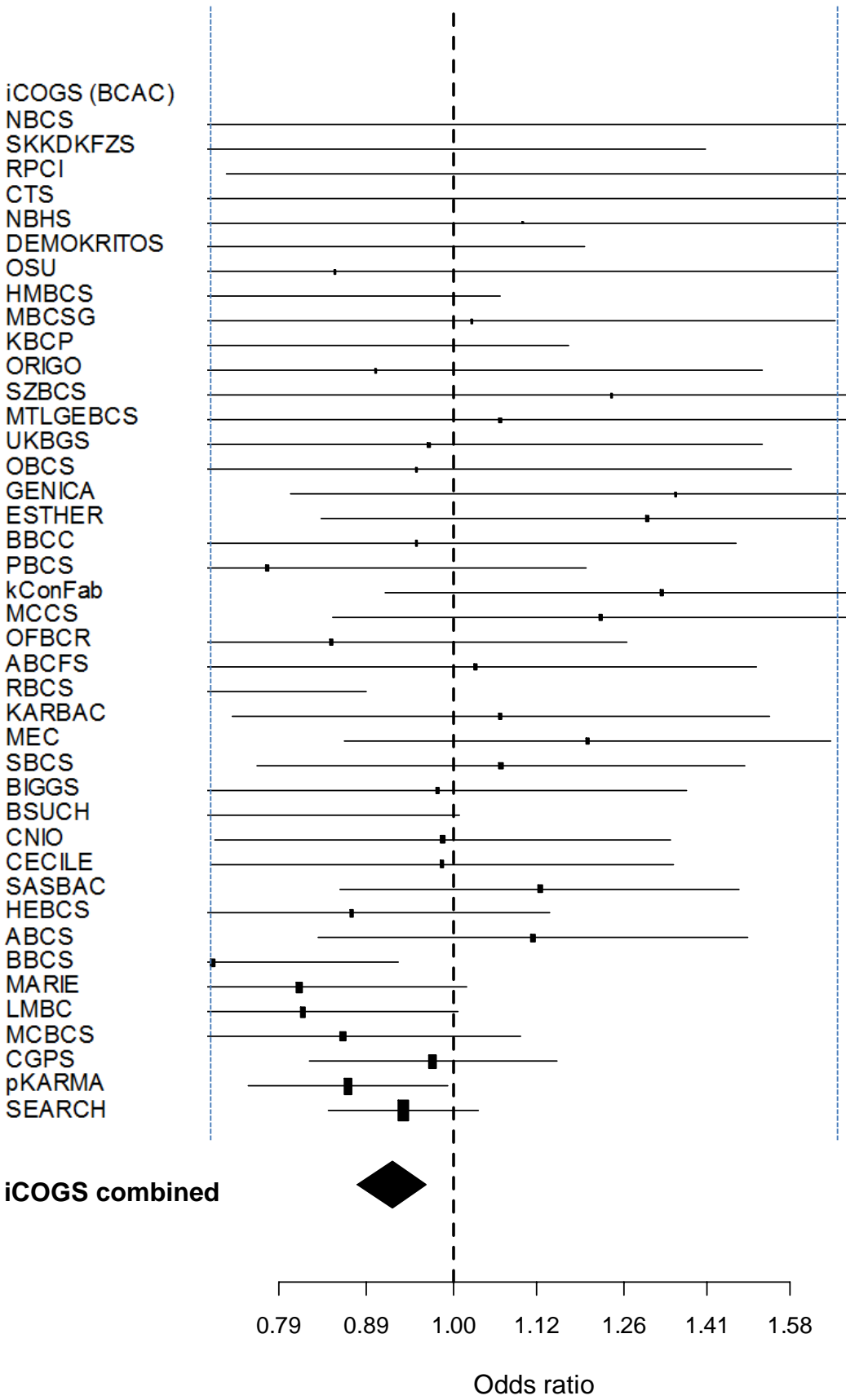

rs1052532

p-het = 0.47

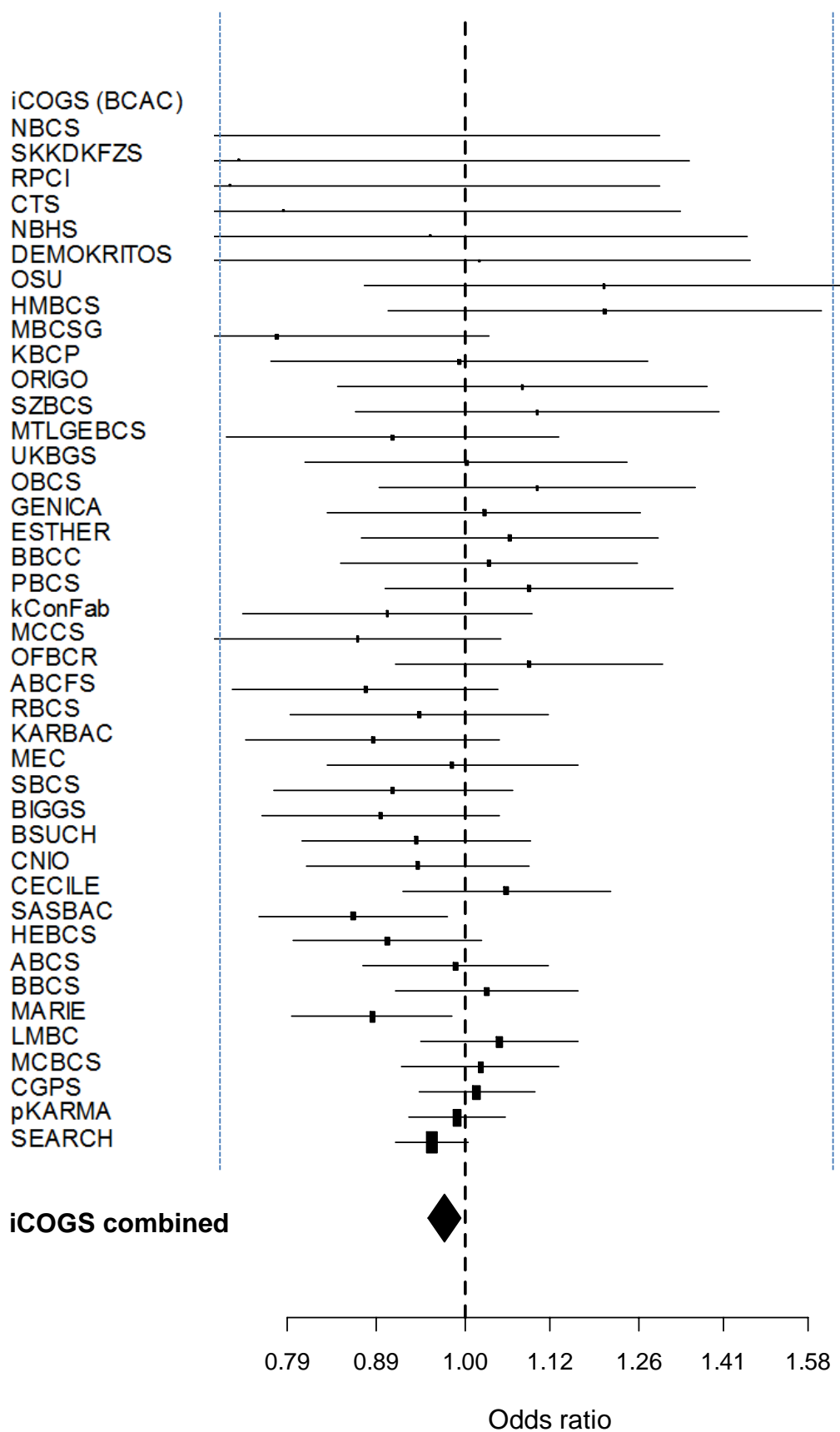

rs10719

p-het = 0.16

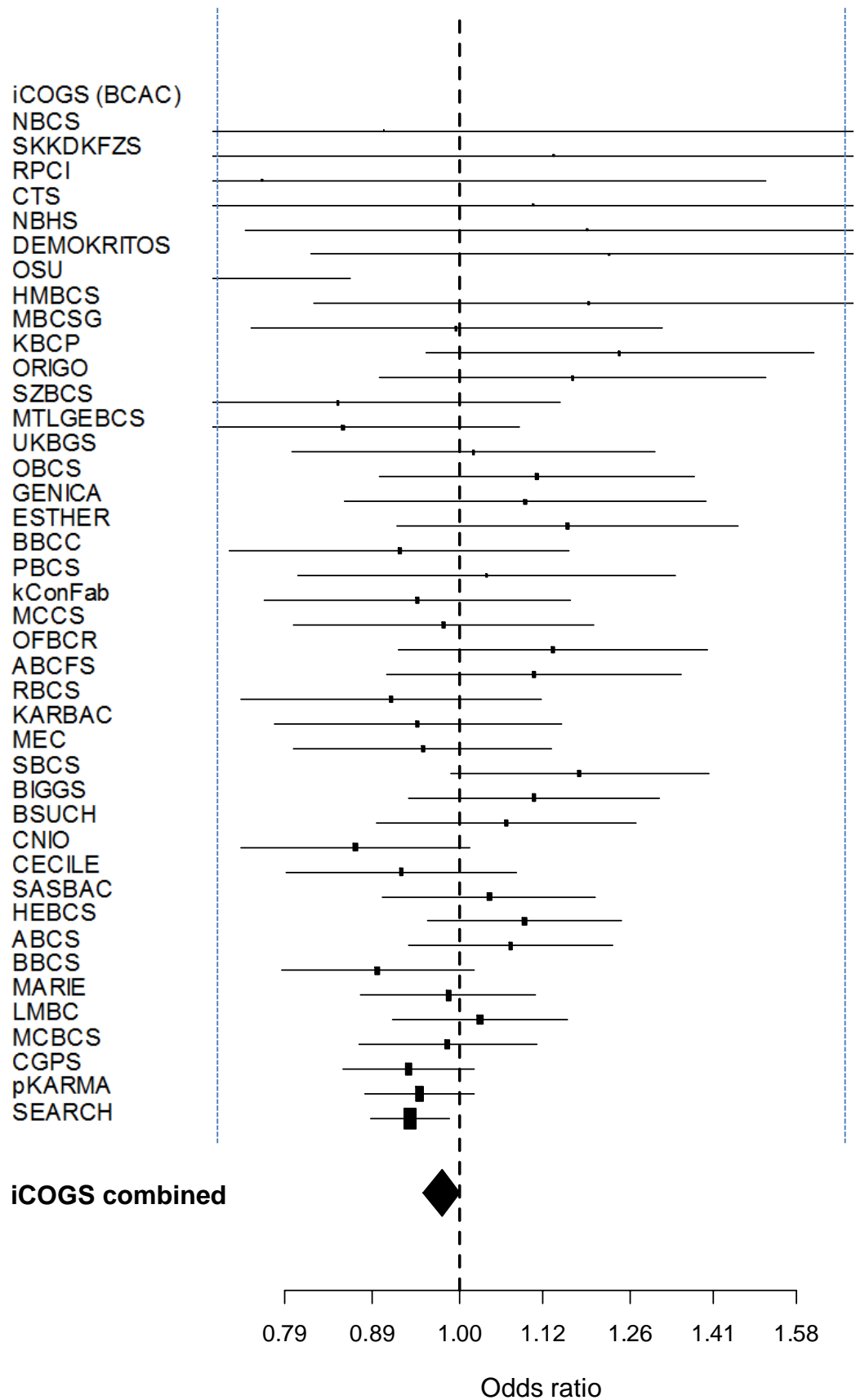

rs4687554

p-het = 0.50

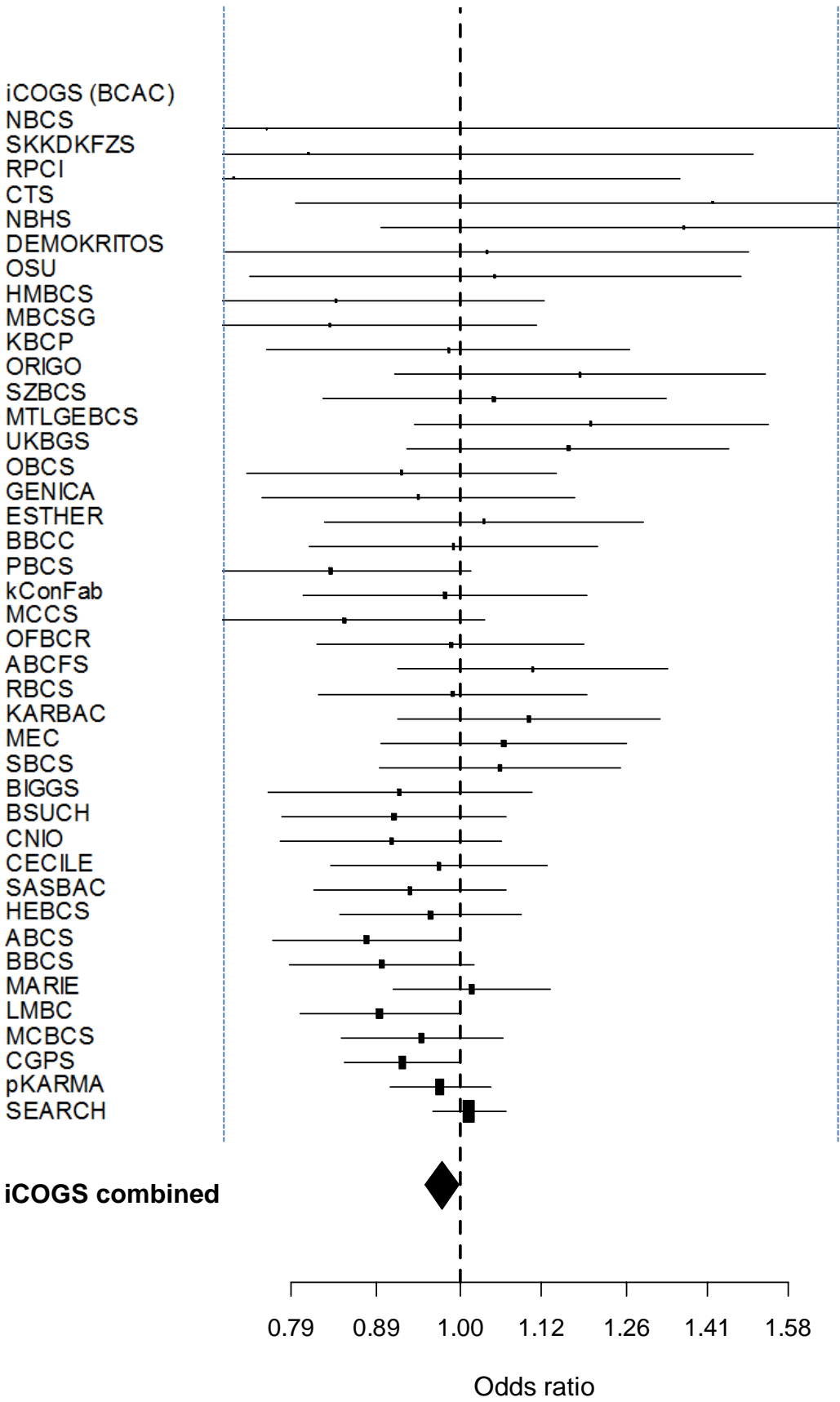

rs3134615

p-het = 0.55

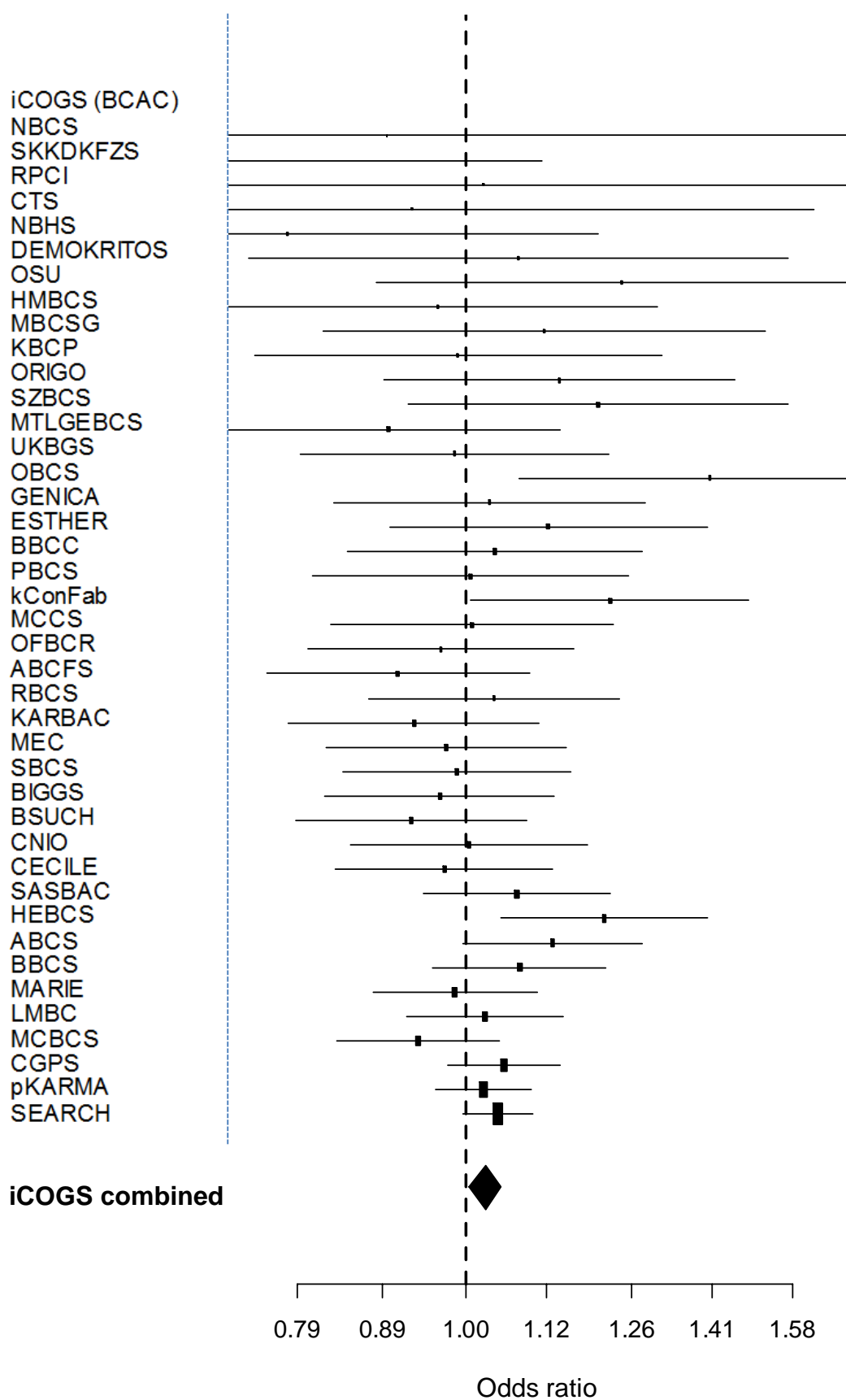

Supplement: Figure S2 — Forest plots for the five most significant miRNA binding site SNPs from the iCOGS. Squares indicate the estimated per-allele OR for the minor allele in Europeans. The horizontal lines indicate 95% confidence limits. The vertical blue dashed lines indicate clipping of the confidence intervals for presentation purpose. The area of the square is inversely proportional to the variance of the estimate. The diamond indicates the estimated per-allele OR from the combined analysis. (PDF) [file pone.0109973.s002.pdf]
