# Supplementary material for: MicroRNA Related Polymorphisms and Breast Cancer Risk
Source: PLoS One. 2014 Nov 12;9(11):e109973. doi: 10.1371/journal.pone.0109973 (PMC4229095; doi:10.1371/journal.pone.0109973)
Supplement: Table S1 — A description of each GWAS study, number of subjects and genotyping platform used in combined GWAS. (DOC) [file pone.0109973.s003.doc]

Table S1. A description of each GWAS study, number of subjects, genotyping platform used in combined GWAS and ethics approval committees.

| **Study abbreviation** | **Full name** | **Country** | **Case ascertainment** | **Control ascertainment** | **Genotyping platform** | **Cases^1^** | **Controls^1^** | **Approval committee** |
| --- | --- | --- | --- | --- | --- | --- | --- | --- |
| ABCFS/  kConFab | Australian Breast Cancer Family Study | Australia | Recruitment through cancer registries in Victoria and New South Wales | Recruitment from the electoral rolls in Melbourne and Sydney matched to cases by age in-5 year categories | Illumina 610k | 282 | 285 | The University of Melbourne Health Sciences Human Ethics Sub-Committee (HESC) |
| BBCS | British Breast Cancer Study | UK | Recruitment through cancer registries and clinics in the UK, predominantly bilateral cases | WTCCC2: 1958 Birth Cohort + UK National Blood Service | Illumina 370k (cases) Illumina 1.2M (controls) | 1609 | 5190 | South East Multi-Centre Research Ethics Committee |
| CGEMS |  | USA | Postmenopausal cases from Nurses Health Study | Individually matched controls from Nurses Health Study | Illumina 550k | 1127 | 1130 | Institutional Review Board of the Brigham and Women’s Hospital |
| DFBBCS | Dutch Familial Bilateral Breast Cancer Study | Netherlands | BRCA1/2 mutation negative familial bilateral breast cancer patients selected from five clinical genetics centers; Erasmus University Medical Center/Daniel den Hoed, The Netherlands Cancer Institute, Leiden University Medical Center, University Medical Center Utrecht, and VU University Medical Center. | Controls were from the Rotterdam study, and are 55 years or older at the time of inclusion. For this study females were selected and breast cancer cases were excluded. | Illumina 610k (cases) Illumina 550k (controls) | 464 | 3255 | Institutional review  board (Medical Ethics Committee) of the Erasmus Medical  Center and the review board of The Netherlands Ministry  of Health, Welfare and Sports |
| GC-HBOC | German Familial Breast Cancer Study | Germany | BRCA1/2 mutation negative cases from University Clinics in Cologne and Munich | KORA (Cooperative Health Research in the Region Augsburg) | Affymetrix 5.0k (cases) Affymetrix 6.0k (controls) | 634 | 477 | Ethik-Kommission der Medizinischen Fakultat der Universitat zu Koln |
| HEBCS | Helsinki Breast Cancer Study | Finland | Unselected cases plus additional familial cases from Helsinki University Central Hospital | Population Controls from from the NordicDB, a Nordic pool and portal for genome-wide control data | Illumina 550k + 610k (cases) Illumina 370k (controls) | 810 | 1012 | Helsingin ja uudenmaan sairaanhoitopiiri (Helsinki University Central Hospital Ethics Committee) |
| MARIE | Mammary Carcinoma Risk Factor Investigation | Germany | Random sample of cases from the MARIE study, but restricted to ductal and lobular carcinomas and oversampled for lobular (about 2:1) | KORA (Cooperative Health Research in the Region Augsburg) | Illumina 370k (cases) Illumina 550k (controls) | 708 | 470 | Ruprecht-Karls-Universitat Medizinische Fakultat Heidelberg Ethikkommission |
| SASBAC | Singapore and Sweden Breast Cancer Study | Sweden | Population based case control study of postmenopausal women | Population based controls frequency matched by age to cases | Illumina 317k+240k (cases) Illumina 550k (controls) | 790 | 756 | Regionala Etikprovningsnamnden i Stockholm (Regional Ethical Review Board in Stockholm) |
| UK2 | UK Familial Breast Cancer Study | UK | UK cancer genetics clinics + oncology clinics | WTCCC2: 1958 Birth Cohort + UK National Blood Service | Illumina 670k (cases) Illumina 1.2M (controls) | 3628 | 5190 | South and West Multi-Centre Research Ethics Committee |

^1^Final numbers used in the analysis, after QC.

**REFERENCES**

1. Dite GS, Jenkins MA, Southey MC, Hocking JS, Giles GG, et al. (2003) Familial risks, early-onset breast cancer, and BRCA1 and BRCA2 germline mutations. J Natl Cancer Inst 95: 448-457.

2. Fletcher O, Johnson N, Palles C, dos Santos Silva I, McCormack V, et al. (2006) Inconsistent association between the STK15 F31I genetic polymorphism and breast cancer risk. J Natl Cancer Inst 98: 1014-1018.

3. Hunter DJ, Kraft P, Jacobs KB, Cox DG, Yeager M, et al. (2007) A genome-wide association study identifies alleles in FGFR2 associated with risk of sporadic postmenopausal breast cancer. Nat Genet 39: 870-874.

4. Hofman A, van Duijn CM, Franco OH, Ikram MA, Janssen HL, et al. (2011) The Rotterdam Study: 2012 objectives and design update. Eur J Epidemiol 26: 657-686.

5. Frank B, Hemminki K, Wappenschmidt B, Meindl A, Klaes R, et al. (2006) Association of the CASP10 V410I variant with reduced familial breast cancer risk and interaction with the CASP8 D302H variant. Carcinogenesis 27: 606-609.

6. Li J, Humphreys K, Heikkinen T, Aittomaki K, Blomqvist C, et al. (2011) A combined analysis of genome-wide association studies in breast cancer. Breast Cancer Res Treat 126: 717-727.

7. Leu M, Humphreys K, Surakka I, Rehnberg E, Muilu J, et al. (2010) NordicDB: a Nordic pool and portal for genome-wide control data. Eur J Hum Genet 18: 1322-1326.

8. Flesch-Janys D, Slanger T, Mutschelknauss E, Kropp S, Obi N, et al. (2008) Risk of different histological types of postmenopausal breast cancer by type and regimen of menopausal hormone therapy. Int J Cancer 123: 933-941.

9. Turnbull C, Ahmed S, Morrison J, Pernet D, Renwick A, et al. (2010) Genome-wide association study identifies five new breast cancer susceptibility loci. Nat Genet 42: 504-507.
