# Supplementary material for: MicroRNA Related Polymorphisms and Breast Cancer Risk
Source: PLoS One. 2014 Nov 12;9(11):e109973. doi: 10.1371/journal.pone.0109973 (PMC4229095; doi:10.1371/journal.pone.0109973)
Supplement: Table S3 — Frequencies and effect sizes of the 42 SNPs in the main analysis; combined GWAS and iCOGS. (DOC) [file pone.0109973.s005.doc]

Table S3. Frequencies and effect sizes of the 42 SNPs in the main analysis; combined GWAS and iCOGS.

| SNP | Chr | Position | coding | Freq^1^ | beta combined GWAS | se combined GWAS | P1df combined GWAS | beta iCOGS | se iCOGS | P1df iCOGS | GENE |
| --- | --- | --- | --- | --- | --- | --- | --- | --- | --- | --- | --- |
| rs702681 | 5 | 56253786 | AG | 0.270 | 0.06456 | 0.0224 | 3.92 x 10^-3^ | 0.06138 | 0.0110 | 2.76 x 10^-8^ | MIER3 |
| rs1045494 | 2 | 201860026 | AG | 0.022 | -0.10376 | 0.0523 | 4.74 x 10^-2^ | -0.08281 | 0.0236 | 4.47 x 10^-4^ | CASP8 |
| rs1052532 | 15 | 89275240 | AG | 0.270 | -0.05923 | 0.0223 | 7.94 x 10^-3^ | -0.02680 | 0.0110 | 1.47 x 10^-2^ | HDDC3 |
| rs3134615 | 1 | 40134653 | CA | 0.261 | 0.03788 | 0.0230 | 9.97 x 10^-2^ | 0.02664 | 0.0115 | 2.09 x 10^-2^ | MYCL1 |
| rs4687554 | 3 | 52839175 | AG | 0.171 | -0.05873 | 0.0235 | 1.23 x 10^-2^ | -0.02626 | 0.0116 | 2.39 x 10^-2^ | MUSTN1 |
| rs10719 | 5 | 31437204 | GA | 0.221 | -0.08260 | 0.0248 | 8.79 x 10^-4^ | -0.02368 | 0.0122 | 5.32 x 10^-2^ | DROSHA |
| rs17512204 | 2 | 118449301 | GA | 0.101 | 0.05805 | 0.0374 | 1.20 x 10^-1^ | 0.02596 | 0.0186 | 1.63 x 10^-1^ | CCDC93 |
| rs7635553 | 3 | 168646064 | GA | 0.025 | -0.11529 | 0.0350 | 9.73 x 10^-4^ | -0.02196 | 0.0171 | 1.98 x 10^-1^ | SERPINI2 |
| rs4351800 | 11 | 7446395 | CA | 0.458 | 0.04084 | 0.0204 | 4.48 x 10^-2^ | 0.01283 | 0.0100 | 1.98 x 10^-1^ | SYT9 |
| rs7086917 | 10 | 49867441 | AC | 0.454 | -0.03690 | 0.0199 | 6.35 x 10^-2^ | -0.00763 | 0.0098 | 4.38 x 10^-1^ | WDFY4 |
| rs7040123 | 9 | 7160742 | AG | 0.024 | 0.09985 | 0.0562 | 7.59 x 10^-2^ | 0.01715 | 0.0263 | 5.14 x 10^-1^ | KDM4C |
| rs3809828 | 17 | 7187575 | GA | 0.046 | 0.15317 | 0.0495 | 1.97 x 10^-3^ | 0.01283 | 0.0201 | 5.22 x 10^-1^ | KCTD11 |
| rs17151639 | 7 | 127425052 | AG | 0.183 | -0.03625 | 0.0226 | 1.09 x 10^-1^ | -0.00648 | 0.0113 | 5.66 x 10^-1^ | SND1 |
| rs3796133 | 3 | 100000533 | GA | 0.030 | 0.16485 | 0.0467 | 4.18 x 10^-4^ | 0.01291 | 0.0229 | 5.74 x 10^-1^ | DCBLD2 |
| rs7441 | 12 | 90063806 | GA | 0.043 | 0.10459 | 0.0399 | 8.70 x 10^-3^ | 0.01026 | 0.0195 | 5.98 x 10^-1^ | DCN |
| rs17480616 | 7 | 134773600 | CG | 0.021 | -0.14461 | 0.0949 | 1.27 x 10^-1^ | -0.01370 | 0.0292 | 6.39 x 10^-1^ | CNOT4 |
| rs7674744 | 4 | 78874296 | GA |  | -0.06235 | 0.0284 | 2.83 x 10^-2^ | -0.00568 | 0.0143 | 6.91 x 10^-1^ | CNOT6L |
| rs2304669 | 2 | 238830402 | AG | 0.124 | -0.03894 | 0.0294 | 1.86 x 10^-1^ | -0.00324 | 0.0140 | 8.17 x 10^-1^ | PER2 |
| rs12438324 | 15 | 55366808 | AG | 0.015 | -0.13509 | 0.0525 | 1.01 x 10^-2^ | -0.00444 | 0.0270 | 8.69 x 10^-1^ | TCF12 |
| rs1058450 | 4 | 120200088 | GA | 0.183 | -0.03944 | 0.0263 | 1.33 x 10^-1^ | -0.00116 | 0.0128 | 9.28 x 10^-1^ | SYNPO2 |
| rs7513934 | 1 | 52590776 | GA | 0.467 | 0.03471 | 0.0198 | 7.98 x 10^-2^ | 0.00001 | 0.0098 | 9.99 x 10^-1^ | CC2D1B |
| Below the SNPs have opposite effect in combined GWAS compared to iCOGS | | | | | | | | | | | |
| rs1444418 | 10 | 64230476 | AG | 0.094 | 0.07012 | 0.0304 | 2.09 x 10^-2^ | -0.05807 | 0.0152 | 1.27 x 10^-4^ | ADO |
| rs2550303 | 16 | 54953111 | AG | 0.391 | -0.05824 | 0.0201 | 3.71 x 10^-3^ | 0.03244 | 0.0099 | 1.04 x 10^-3^ | AMFR |
| rs4705870 | 5 | 132187033 | GA | 0.182 | 0.05309 | 0.0301 | 7.79 x 10^-2^ | -0.02030 | 0.0137 | 1.39 x 10^-1^ | ANKRD43 |
| rs2037119 | 3 | 63969919 | GA | 0.346 | -0.05536 | 0.0216 | 1.03 x 10^-2^ | 0.03671 | 0.0107 | 5.67 x 10^-4^ | ATXN7 |
| rs8140217 | 22 | 37547947 | GA | 0.200 | -0.04983 | 0.0253 | 4.88 x 10^-2^ | 0.00165 | 0.0129 | 8.98 x 10^-1^ | CBX6 |
| rs1128226 | 7 | 21908194 | AC | 0.370 | 0.06608 | 0.0210 | 1.62 x 10^-3^ | -0.03973 | 0.0102 | 9.32 x 10^-5^ | CDCA7L |
| rs1803439 | 21 | 37807312 | AG | 0.382 | -0.03553 | 0.0212 | 9.37 x 10^-2^ | 0.00884 | 0.0103 | 3.91 x 10^-1^ | DYRK1A |
| rs3797 | 15 | 27199858 | AG | 0.061 | 0.09440 | 0.0461 | 4.05 x 10^-2^ | -0.02217 | 0.0214 | 3.01 x 10^-1^ | FAM189A1 |
| rs7130622 | 11 | 128186721 | AC | 0.006 | 0.27933 | 0.3885 | 4.72 x 10^-1^ | -0.34704 | 0.2265 | 1.25 x 10^-1^ | FLI1 |
| rs9473 | 10 | 13727177 | GA | 0.415 | -0.03661 | 0.0201 | 6.83 x 10^-2^ | 0.00790 | 0.0099 | 4.27 x 10^-1^ | FRMD4A |
| rs17091403 | 10 | 115923895 | GA | 0.098 | 0.06234 | 0.0353 | 7.78 x 10^-2^ | -0.01276 | 0.0161 | 4.29 x 10^-1^ | hsa-miR^-2^110 |
| rs1805360 | 11 | 93866677 | GA | 0.006 | -0.15546 | 0.1061 | 1.43 x 10^-1^ | 0.06421 | 0.0523 | 2.20 x 10^-1^ | hsa-mir^-5^48l |
| rs1062225 | 10 | 49313232 | AG | 0.112 | 0.06101 | 0.0308 | 4.80 x 10^-2^ | -0.04953 | 0.0146 | 7.22 x 10^-4^ | MAPK8 |
| rs41739 | 7 | 116224740 | AG | 0.458 | -0.06749 | 0.0200 | 7.38 x 10^-4^ | 0.00506 | 0.0099 | 6.10 x 10^-1^ | MET |
| rs9371201 | 6 | 150186694 | GA | 0.318 | -0.03445 | 0.0207 | 9.62 x 10^-2^ | 0.01372 | 0.0103 | 1.84 x 10^-1^ | PCMT1 |
| rs13422 | 17 | 15074900 | AC | 0.455 | -0.04059 | 0.0197 | 3.92 x 10^-2^ | 0.02478 | 0.0098 | 1.15 x 10^-2^ | PMP22 |
| rs7562391 | 2 | 201444411 | AC | 0.136 | 0.07076 | 0.0300 | 1.85 x 10^-2^ | -0.03530 | 0.0149 | 1.80 x 10^-2^ | PPIL3 |
| rs7520333 | 1 | 40862837 | AG | 0.461 | -0.05261 | 0.0197 | 7.70 x 10^-3^ | 0.00729 | 0.0100 | 4.66 x 10^-1^ | RIMS3 |
| rs3764941 | 5 | 135497426 | AC | 0.300 | 0.04950 | 0.0216 | 2.22 x 10^-2^ | -0.01781 | 0.0108 | 9.78 x 10^-2^ | SMAD5 |
| rs739692 | 18 | 53178524 | GA | 0.142 | 0.05841 | 0.0268 | 2.93 x 10^-2^ | -0.02356 | 0.0131 | 7.12 x 10^-2^ | ST8SIA3 |
| rs12869870 | 13 | 99415306 | GA | 0.197 | 0.03905 | 0.0277 | 1.58 x 10^-1^ | -0.01601 | 0.0117 | 1.71 x 10^-1^ | ZIC5 |

^1^Minor allele frequency in Caucasian population in Hapmap.
