# Supplementary material for: MicroRNA Related Polymorphisms and Breast Cancer Risk
Source: PLoS One. 2014 Nov 12;9(11):e109973. doi: 10.1371/journal.pone.0109973 (PMC4229095; doi:10.1371/journal.pone.0109973)
Supplement: Table S4 — Results for SNPs in the GWAS and iCOGS separately and combined GWAS+iCOGS analysis for ER negative subgroup. (DOC) [file pone.0109973.s006.doc]

Table S4. Results for SNPs in the GWAS and iCOGS separately and combined GWAS+iCOGS analysis for ER negative subgroup.

| SNP | Chr | Position | coding^1^ | GWAS OR (95%CI)^2^ | GWAS *P*^3^ | iCOGS OR (95% CI)^2^ | iCOGS *P*^3^ | Combined GWAS+iCOGS OR (95% CI)^2^ | Combined GWAS+iCOGS *P*^3^ (BH corrected *P*)^4^ | Gene |
| --- | --- | --- | --- | --- | --- | --- | --- | --- | --- | --- |
| rs3809828 | 17 | 7187575 | CT | 1,14 (0,90 - 1,43) | 2.88 x 10^-1^ | 1,09 (1,01 - 1,18) | 3.27 x 10^-2^ | 1,09 (1,02 - 1,18) | 1.81 x 10^-2^ (3.99 x 10^-1^) | KCTD11 |
| rs4687554 | 3 | 52839175 | CT | 0,97 (0,86 - 1,10) | 6.63 x 10^-1^ | 0,95 (0,90 - 1,00) | 3.54 x 10^-2^ | 0,95 (0,91 - 1,00) | 3.39 x 10^-2^ (3.99 x 10^-1^) | MUSTN1 |
| rs1052532 | 15 | 89275240 | CT | 0,95 (0,84 - 1,07) | 4.02 x 10^-1^ | 0,96 (0,92 - 1,00) | 6.32 x 10^-2^ | 0,96 (0,92 - 1,00) | 4.21 x 10^-2^ (3.99 x 10^-1^) | HDDC3 |
| rs4351800 | 11 | 7446395 | AC | 1,08 (0,96 - 1,21) | 1.90 x 10^-1^ | 1,03 (0,99 - 1,07) | 1.79 x 10^-1^ | 1,03 (1,00 - 1,07) | 8.75 x 10^-2^ (4.87 x 10^-1^) | SYT9 |
| rs10719 | 5 | 31437204 | AG | 0,86 (0,75 - 0,98) | 2.81 x 10^-2^ | 0,98 (0,93 - 1,03) | 3.68 x 10^-1^ | 0,96 (0,92 - 1,01) | 1.07 x 10^-1^ (4.91 x 10^-1^) | DROSHA |
| rs1045494 | 2 | 201860026 | CT | 0,97 (0,73 - 1,28) | 8.14 x 10^-1^ | 0,92 (0,84 - 1,02) | 1.14 x 10^-1^ | 0,93 (0,85 - 1,02) | 1.17 x 10^-1^ (4.91 x 10^-1^) | CASP8 |
| rs3134615 | 1 | 40134653 | AC | 1,12 (0,98 - 1,28) | 9.33 x 10^-2^ | 1,02 (0,98 - 1,07) | 3.32 x 10^-1^ | 1,03 (0,99 - 1,08) | 1.39 x 10^-1^ (5.31 x 10^-1^) | MYCL1 |
| rs7441 | 12 | 90063806 | AG | 1,05 (0,82 - 1,33) | 7.12 x 10^-1^ | 1,05 (0,97 - 1,14) | 2.02 x 10^-1^ | 1,05 (0,98 - 1,13) | 1.84 x 10^-1^ (5.52 x 10^-1^) | DCN |
| rs7086917 | 10 | 49867441 | GT | 0,90 (0,81 - 1,01) | 7.90 x 10^-2^ | 0,99 (0,95 - 1,03) | 6.37 x 10^-1^ | 0,98 (0,94 - 1,02) | 2.98 x 10^-1^ (6.94 x 10^-1^) | WDFY4 |
| rs3796133 | 3 | 100000533 | AG | 1,07 (0,83 - 1,40) | 5.91 x 10^-1^ | 1,03 (0,94 - 1,13) | 5.44 x 10^-1^ | 1,03 (0,95 - 1,13) | 4.53 x 10^-1^ (8.21 x 10^-1^) | DCBLD2 |
| rs17151639 | 7 | 127425052 | AG | 0,79 (0,70 - 0,91) | 5.47 x 10^-4^ | 1,01 (0,97 - 1,06) | 6.15 x 10^-1^ | 0,99 (0,94 - 1,03) | 4.98 x 10^-1^ (8.21 x 10^-1^) | SND1 |
| rs12438324 | 15 | 55366808 | AG | 0,94 (0,69 - 1,26) | 6.66 x 10^-1^ | 0,97 (0,87 - 1,09) | 6.08 x 10^-1^ | 0,97 (0,87 - 1,07) | 5.28 x 10^-1^ (8.21 x 10^-1^) | TCF12 |
| rs1058450 | 4 | 120200088 | CT | 1,09 (0,94 - 1,27) | 2.70 x 10^-1^ | 1,00 (0,95 - 1,06) | 9.03 x 10^-1^ | 1,01 (0,96 - 1,06) | 6.32 x 10^-1^ (8.32 x 10^-1^) | SYNPO2 |
| rs7513934 | 1 | 52590776 | AG | 1,06 (0,95 - 1,18) | 3.18 x 10^-1^ | 1,00 (0,96 - 1,04) | 9.77 x 10^-1^ | 1,01 (0,97 - 1,04) | 7.56 x 10^-1^ (9.07 x 10^-1^) | CC2D1B |
| rs2304669 | 2 | 238830402 | CT | 1,01 (0,86 - 1,18) | 9.17 x 10^-1^ | 1,00 (0,94 - 1,06) | 9.36 x 10^-1^ | 1,00 (0,95 - 1,05) | 9.68 x 10^-1^ (9.68 x 10^-1^) | PER2 |

^1^Build 36 position

^2^Per allele odds ratio for the minor allele relative to the major allele

^3^1df p-trend

^4^1df p-trend adjusted against multiple testing by Benjamini–Hochberg correction method
