# Supplementary material for: MicroRNA Related Polymorphisms and Breast Cancer Risk
Source: PLoS One. 2014 Nov 12;9(11):e109973. doi: 10.1371/journal.pone.0109973 (PMC4229095; doi:10.1371/journal.pone.0109973)
Supplement: Table S5 — Results for SNPs in the GWAS and iCOGS separately and combined GWAS+iCOGS analysis for ER positive subgroup. (DOC) [file pone.0109973.s007.doc]

Table S5. Results for SNPs in the GWAS and iCOGS separately and combined GWAS+iCOGS analysis for ER positive subgroup.

| SNP | Chr | Position | coding^1^ | GWAS OR (95%CI)^2^ | GWAS *P*^3^ | iCOGS OR (95% CI)^2^ | iCOGS *P*^3^ | Combined GWAS+iCOGS OR (95% CI)^2^ | Combined GWAS+iCOGS *P*^3^ (BH corrected *P*)^4^ | Gene |
| --- | --- | --- | --- | --- | --- | --- | --- | --- | --- | --- |
| rs702681 | 5 | 56253786 | AG | 1.07 (1.04 - 1.09) | 2.62 x 10^-7^ | 1.07 (1.04 - 1.09) | 9.20 x 10^-7^ | 1.07 (1.05 - 1.09) | 1.16 x 10^-12^ (2.44 x 10^-11^) | MIER3 |
| rs1045494 | 2 | 201860026 | AG | 0.92 (0.88 - 0.97) | 3.25 x 10^-3^ | 0.92 (0.87 - 0.98) | 4.68 x 10^-3^ | 0.92 (0.89 - 0.96) | 4.51 x 10^-5^ (4.74 x 10^-4^) | CASP8 |
| rs4687554 | 3 | 52839175 | AG | 0.96 (0.94 - 0.99) | 5.65 x 10^-3^ | 0.97 (0.94 - 0.99) | 1.24 x 10^-2^ | 0.97 (0.95 - 0.98) | 1.95 x 10^-4^ (1.37 x 10^-3^) | MUSTN1 |
| rs3134615 | 1 | 40134653 | CA | 1.03 (1 - 1.06) | 2.63 x 10^-2^ | 1.04 (1.01 - 1.06) | 1.08 x 10^-2^ | 1.03 (1.01 - 1.05) | 7.75 x 10^-4^ (4.07 x 10^-3^) | MYCL1 |
| rs17512204 | 2 | 118449301 | GA | 1.04 (1 - 1.09) | 4.71 x 10^-2^ | 1.03 (0.98 - 1.07) | 2.62 x 10^-1^ | 1.03 (1 - 1.07) | 2.68 x 10^-2^ (1.13 x 10^-1^) | CCDC93 |
| rs10719 | 5 | 31437204 | GA | 0.97 (0.95 - 1) | 6.14 x 10^-2^ | 0.99 (0.96 - 1.02) | 3.56 x 10^-1^ | 0.98 (0.96 - 1) | 4.69 x 10^-2^ (1.64 x 10^-1^) | DROSHA |
| rs1058450 | 4 | 120200088 | GA | 0.98 (0.96 - 1.01) | 2.73 x 10^-1^ | 0.99 (0.96 - 1.02) | 3.46 x 10^-1^ | 0.98 (0.96 - 1.01) | 1.48 x 10^-1^ (3.74 x 10^-1^) | SYNPO2 |
| rs7040123 | 9 | 7160742 | AG | 1.02 (0.97 - 1.09) | 4.05 x 10^-1^ | 1.04 (0.97 - 1.10) | 2.65 x 10^-1^ | 1.03 (0.99 - 1.07) | 1.70 x 10^-1^ (3.74 x 10^-1^) | KDM4C |
| rs1052532 | 15 | 89275240 | AG | 0.99 (0.96 - 1.01) | 2.86 x 10^-1^ | 0.99 (0.96 - 1.01) | 4.01 x 10^-1^ | 0.99 (0.97 - 1.01) | 1.75 x 10^-1^ (3.74 x 10^-1^) | HDDC3 |
| rs7441 | 12 | 90063806 | GA | 1.03 (0.99 - 1.08) | 1.78 x 10^-1^ | 1.01 (0.97 - 1.06) | 5.90 x 10^-1^ | 1.02 (0.99 - 1.05) | 1.78 x 10^-1^ (3.74 x 10^-1^) | DCN |
| rs4351800 | 11 | 7446395 | CA | 1.01 (0.99 - 1.03) | 2.94 x 10^-1^ | 1.01 (0.98 - 1.03) | 6.24 x 10^-1^ | 1.01 (0.99 - 1.03) | 2.70 x 10^-1^ (5.10 x 10^-1^) | SYT9 |
| rs7635553 | 3 | 168646064 | GA | 0.99 (0.95 - 1.02) | 4.54 x 10^-1^ | 0.99 (0.95 - 1.03) | 5.00 x 10^-1^ | 0.99 (0.96 - 1.01) | 3.13 x 10^-1^ (5.10 x 10^-1^) | SERPINI2 |
| rs7513934 | 1 | 52590776 | GA | 1.01 (0.99 - 1.03) | 5.53 x 10^-1^ | 1.01 (0.99 - 1.03) | 4.04 x 10^-1^ | 1.01 (0.99 - 1.02) | 3.16 x 10^-1^ (5.10 x 10^-1^) | CC2D1B |
| rs3809828 | 17 | 7187575 | GA | 1.01 (0.97 - 1.06) | 6.55 x 10^-1^ | 1.00 (0.96 - 1.05) | 8.50 x 10^-1^ | 1.01 (0.98 - 1.04) | 6.48 x 10^-1^ (9.21 x 10^-1^) | KCTD11 |
| rs3796133 | 3 | 100000533 | GA | 1.01 (0.96 - 1.07) | 6.01 x 10^-1^ | 1.00 (0.95 - 1.06) | 9.53 x 10^-1^ | 1.01 (0.97 - 1.05) | 6.74 x 10^-1^ (9.21 x 10^-1^) | DCBLD2 |
| rs2304669 | 2 | 238830402 | AG | 1 (0.97 - 1.03) | 9.98 x 10^-1^ | 1.01 (0.97 - 1.04) | 7.13 x 10^-1^ | 1 (0.98 - 1.03) | 8.01 x 10^-1^ (9.21 x 10^-1^) | PER2 |
| rs17480616 | 7 | 134773600 | CG | 1 (0.93 - 1.07) | 9.52 x 10^-1^ | 0.99 (0.93 - 1.06) | 7.86 x 10^-1^ | 0.99 (0.95 - 1.04) | 8.15 x 10^-1^ (9.21 x 10^-1^) | CNOT4 |
| rs17151639 | 7 | 127425052 | AG | 1 (0.97 - 1.02) | 9.49 x 10^-1^ | 1.00 (0.97 - 1.02) | 8.78 x 10^-1^ | 1 (0.98 - 1.02) | 8.80 x 10^-1^ (9.21 x 10^-1^) | SND1 |
| rs7086917 | 10 | 49867441 | AC | 1 (0.98 - 1.02) | 8.91 x 10^-1^ | 1.00 (0.98 - 1.02) | 9.99 x 10^-1^ | 1 (0.99 - 1.02) | 9.21 x 10^-1^ (9.21 x 10^-1^) | WDFY4 |

^1^Build 36 position

^2^ Per allele odds ratio for the minor allele relative to the major allele

^3^1df p-trend

^4^1df p-trend adjusted against multiple testing by Benjamini–Hochberg correction method
