# Supplementary material for: MicroRNA Related Polymorphisms and Breast Cancer Risk
Source: PLoS One. 2014 Nov 12;9(11):e109973. doi: 10.1371/journal.pone.0109973 (PMC4229095; doi:10.1371/journal.pone.0109973)
Supplement: Table S6 — Results for SNPs in the GWAS and iCOGS separately and combined GWAS+iCOGS analysis for cases less than 50 years at diagnosis. (DOC) [file pone.0109973.s008.doc]

Table S6. Results for SNPs in the GWAS and iCOGS separately and combined GWAS+iCOGS analysis for cases less than 50 years at diagnosis.

| SNP | Chr | Position | coding^1^ | GWAS OR (95%CI)^2^ | GWAS *P*^3^ | iCOGS OR (95% CI)^2^ | iCOGS *P*^3^ | Combined GWAS+iCOGS OR (95% CI)^2^ | Combined GWAS+  iCOGS *P*^3^ (BH corrected P)^4^ | Gene |
| --- | --- | --- | --- | --- | --- | --- | --- | --- | --- | --- |
| rs4351800 | 11 | 7446395 | AC | 1,09 (1,02 - 1,15) | 5.67 x 10^-3^ | 1,04 (1,00 - 1,08) | 3.79 x 10^-2^ | 1,05 (1,02 - 1,09) | 1.26 x 10^-3^ (5.29 x 10^-2^) | SYT9 |
| rs702681 | 5 | 56253786 | CT | 1,06 (0,99 - 1,13) | 9.63 x 10^-2^ | 1,05 (1,00 - 1,09) | 3.03 x 10^-2^ | 1,05 (1,01 - 1,09) | 6.61 x 10^-3^ (9.24 x 10^-2^) | MIER3 |
| rs1045494 | 2 | 201860026 | CT | 0,90 (0,77 - 1,04) | 1.59 x 10^-1^ | 0,90 (0,83 - 0,99) | 2.77 x 10^-2^ | 0,90 (0,84 - 0,97) | 9.02 x 10^-3^ (9.24 x 10^-2^) | CASP8 |
| rs4687554 | 3 | 52839175 | CT | 0,97 (0,90 - 1,04) | 3.53 x 10^-1^ | 0,95 (0,91 - 0,99) | 1.57 x 10^-2^ | 0,95 (0,92 - 0,99) | 1.10 x 10^-2^ (9.24 x 10^-2^) | MUSTN1 |
| rs17512204 | 2 | 118449301 | AG | 1,14 (1,03 - 1,27) | 1.36 x 10^-2^ | 1,02 (0,95 - 1,09) | 5.64 x 10^-1^ | 1,05 (1,00 - 1,12) | 7.05 x 10^-2^ (4.03 x 10^-1^) | CCDC93 |
| rs7441 | 12 | 90063806 | AG | 1,13 (1,01 - 1,27) | 3.60 x 10^-2^ | 1,03 (0,96 - 1,11) | 4.31 x 10^-1^ | 1,06 (0,99 - 1,12) | 7.68 x 10^-2^ (4.03 x 10^-1^) | DCN |
| rs10719 | 5 | 31437204 | AG | 0,94 (0,88 - 1,01) | 1.14 x 10^-1^ | 0,98 (0,94 - 1,03) | 3.77 x 10^-1^ | 0,97 (0,93 - 1,01) | 1.12 x 10^-1^ (4.08 x 10^-1^) | DROSHA |
| rs17151639 | 7 | 127425052 | AG | 0,94 (0,88 - 1,00) | 5.42 x 10^-2^ | 0,99 (0,95 - 1,03) | 5.12 x 10^-1^ | 0,97 (0,94 - 1,01) | 1.13 x 10^-1^ (4.08 x 10^-1^) | SND1 |
| rs7040123 | 9 | 7160742 | AG | 1,24 (1,05 - 1,47) | 1.35 x 10^-2^ | 1,02 (0,92 - 1,12) | 7.22 x 10^-1^ | 1,07 (0,98 - 1,16) | 1.22 x 10^-1^ (4.08 x 10^-1^) | KDM4C |
| rs3809828 | 17 | 7187575 | CT | 1,13 (0,98 - 1,31) | 8.66 x 10^-2^ | 1,03 (0,95 - 1,11) | 4.81 x 10^-1^ | 1,05 (0,98 - 1,12) | 1.57 x 10^-1^ (4.40 x 10^-1^) | KCTD11 |
| rs3796133 | 3 | 100000533 | AG | 1,18 (1,02 - 1,35) | 2.29 x 10^-2^ | 1,01 (0,93 - 1,10) | 8.52 x 10^-1^ | 1,05 (0,98 - 1,13) | 1.82 x 10^-1^ (4.78 x 10^-1^) | DCBLD2 |
| rs1052532 | 15 | 89275240 | CT | 0,98 (0,92 - 1,04) | 4.79 x 10^-1^ | 0,98 (0,94 - 1,02) | 3.90 x 10^-1^ | 0,98 (0,95 - 1,02) | 2.69 x 10^-1^ (5.37 x 10^-1^) | HDDC3 |
| rs17480616 | 7 | 134773600 | CG | 0,94 (0,72 - 1,22) | 6.38 x 10^-1^ | 0,95 (0,85 - 1,06) | 3.46 x 10^-1^ | 0,95 (0,86 - 1,05) | 2.93 x 10^-1^ (5.37 x 10^-1^) | CNOT4 |
| rs3134615 | 1 | 40134653 | AC | 1,00 (0,94 - 1,07) | 9.85 x 10^-1^ | 1,03 (0,98 - 1,07) | 2.30 x 10^-1^ | 1,02 (0,98 - 1,06) | 3.07 x 10^-1^ (5.37 x 10^-1^) | MYCL1 |
| rs7513934 | 1 | 52590776 | AG | 1,05 (0,99 - 1,11) | 1.15 x 10^-1^ | 1,00 (0,97 - 1,04) | 9.41 x 10^-1^ | 1,01 (0,98 - 1,05) | 3.69 x 10^-1^ (5.96 x 10^-1^) | CC2D1B |

^1^Build 36 position

^2^ Per allele odds ratio for the minor allele relative to the major allele

^3^1df p-trend

^4^1df p-trend adjusted against multiple testing by Benjamini–Hochberg correction method
